# PI3K/AKT activation induces PTEN ubiquitination and destabilization accelerating tumourigenesis

**DOI:** 10.1038/ncomms8769

**Published:** 2015-07-17

**Authors:** Min-Sik Lee, Man-Hyung Jeong, Hyun-Woo Lee, Hyun-Ji Han, Aram Ko, Stephen M. Hewitt, Jae-Hoon Kim, Kyung-Hee Chun, Joon-Yong Chung, Cheolju Lee, Hanbyoul Cho, Jaewhan Song

**Affiliations:** 1 Department of Biochemistry, College of Life Science and Biotechnology, Yonsei University, Seoul 120-749, Republic of Korea; 2 Department of Biochemistry and Molecular Biology, Yonsei University College of Medicine, Seoul 120-752, Republic of Korea; 3 Experimental Pathology Laboratory, Center for Cancer Research, National Cancer Institute, NIH MSC 1500, Bethesda, Maryland 20892, USA; 4 Department of Obstetrics and Gynecology, Gangnam Severance Hospital, Yonsei University College of Medicine, Seoul 135-720, Republic of Korea; 5 Institute of Women's Life Medical Science, Yonsei University College of Medicine, Seoul 120-752, Republic of Korea; 6 BRI, Korea Institute of Science and Technology, Seoul 136-791, Korea

## Abstract

The activity of the phosphatase and tensin homologue (PTEN) is known to be suppressed via post-translational modification. However, the mechanism and physiological significance by which post-translational modifications lead to PTEN suppression remain unclear. Here we demonstrate that PTEN destabilization is induced by EGFR- or oncogenic PI3K mutation-mediated AKT activation in cervical cancer. EGFR/PI3K/AKT-mediated ubiquitination and degradation of PTEN are dependent on the MKRN1 E3 ligase. These processes require the stabilization of MKRN1 via AKT-mediated phosphorylation. In cervical cancer patients with high levels of pAKT and MKRN1 expression, PTEN protein levels are low and correlate with a low 5-year survival rate. Taken together, our results demonstrate that PI3K/AKT signals enforce positive-feedback regulation by suppressing PTEN function.

## Abstract (web summary)

Mutations and post-translational modifications of the PI3K/AKT pathway inhibitor PTEN are a feature of many cancers, but these have not been associated with cervical cancer. Here, the authors identify a PI3K/AKT-mediated ubiquitination degradation pathway of PTEN that occurs in patients with cervical cancer.

## Graphical abstract

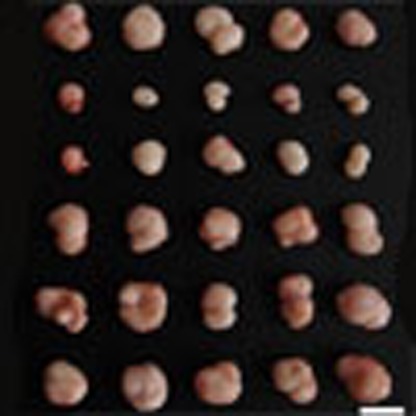

A potent inhibitor of the PI3K/AKT pathway is PTEN (phosphatase and tensin homologue), a tumour suppressor that is highly mutated or deleted in various human cancers^1^,^2^,^3^,^4^. PTEN plays a pivotal role in tumour cell growth and migration^5^,^6^,^7^. The germline mutation of PTEN in several inherited cancer syndromes such as Cowden syndrome, Bannayan–Zonana syndrome and Lhermitte–Duclos disease supports the importance of PTEN as a tumour suppressor^8^,^9^,^10^. Although somatic mutation or genetic deletion of PTEN in cervical cancers has been reported previously, a tumour-suppressing role of PTEN in cervical carcinoma has yet to be identified^11^. Functionally, the lipid phosphatase PTEN antagonizes the PI3K–AKT signalling pathway by catalysing phosphatidylinositol-3,4,5-trisphosphate (PIP3) to phosphatidylinositol-4,5-bisphosphate (PIP2)^6^. Thus, PTEN exhibits tumour-suppressing abilities by inactivating downstream oncogenic AKT-mediated signalling. In addition to genetic alterations of PTEN in human cancers, various post-translational modifications (PTMs) of PTEN have been actively investigated, including phosphorylation, ubiquitination, acetylation and oxidation; these PTMs control the ability of PTEN to inhibit PI3K/AKT signalling^7^,^12^,^13^,^14^. Frequent genetic aberrations of PTEN in human cancer, including depletion of the PTEN protein, are observed in only 25% of cancers^15^, suggesting that PTMs may be important in PTEN function. The recent discovery of several E3 ligases and deubiquitinases such as NEDD4-1, WWP2, XIAP, CHIP, SPOP, USP7 (HAUSP) and USP13 seems to imply that PTEN stability and subcellular localization are important in cancer development^14^,^16^,^17^,^18^,^19^,^20^,^21^. However, the physiological context of PTEN stability has yet to be addressed.

MKRN1 has been identified as an oncogenic E3 ligase that induces the degradation of p53, p21, p14ARF and FADD activities^22^,^23^,^24^,^25^. While MKRN1 has been proposed to function as an inhibitor of tumour suppressors, the upstream pathways regulating MKRN1 function have yet to be identified. Here, we demonstrate that a growth factor-activated PI3K/AKT pathway suppresses PTEN via MKRN1-mediated ubiquitination and degradation. The positive relationship between these post-translational regulatory pathways and clinical evidence is confirmed by analysis of samples from cervical cancer patients. Supporting these observations, xenografts and biochemical analyses indicate inverse correlations between PTEN and the PI3K/AKT-activated MKRN1 axis. These findings suggest a pathway for PI3K/AKT signalling in the regulation of PTEN.

## Results

### AKT signalling is suppressed following depletion of MKRN1

**Figure 1:**
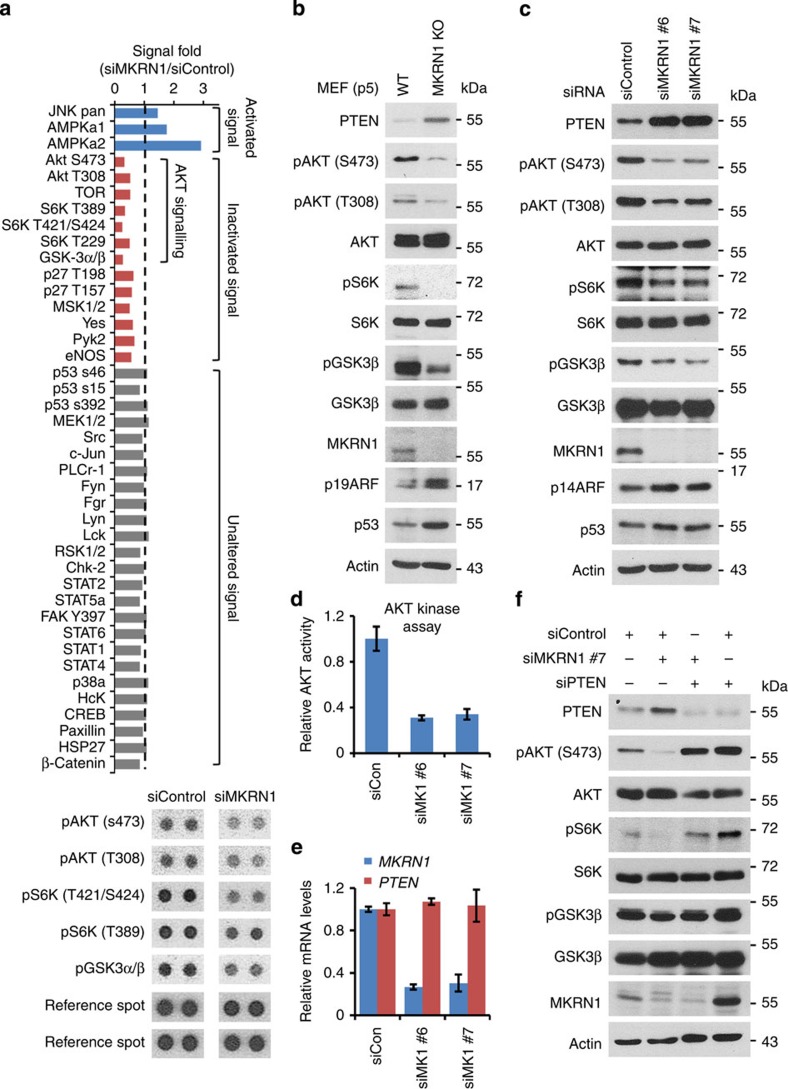
Depletion of MKRN1 suppresses the AKT signalling pathway. (**a**) Alteration of cellular signalling in an MKRN-abrogated cervical cell line. HeLa cells were transduced with 20 nM control siRNA (siControl) or MKRN1 siRNA #7 (siMKRN1) and harvested 48 h post transfection. The diluted lysates were analysed using a human phosphokinase array kit (R&D Systems, ARY003). The mean values (*n*=2 in each blot) of phosphoproteins quantified relative to control siRNA-transfected cells (siControl) were divided into three groups (blue: activated signal, red: inactivated signal and grey: unaltered signal). (**b,c**) MKRN1 depletion suppresses the AKT signalling pathway and elevates PTEN expression. Wild-type (WT) or MKRN1 knockout (MKRN1 KO) mouse embryonic fibroblasts (MEFs) were analysed by immunoblotting at passage 5. RNAi knockdown was performed in HeLa cells with two independent siRNAs for MKRN1 (siMKRN1 #6 and siMKRN1 #7). (**d**) MKRN1 RNAi inhibits AKT activity *in vitro*. *In vitro* AKT kinase assay were performed using AKT purified from siControl or siMKRN1-transfected HeLa cells. Error bars indicate s.d. *n*=3. (**e**) MKRN1 RNAi had no effect on the mRNA level of PTEN. Total RNA was purified from siControl or the two types of siMKRN1-transfected HeLa cells, and mRNA levels of MKRN1 and PTEN were validated by real-time PCR analysis (data shown as mean±s.d.; *n*=3). (**f**) MKRN1 knockdown inhibits AKT signalling in a PTEN-dependent manner. HeLa cells were transduced with RNAi for MKRN1 #7 or PTEN as indicated. Cells were lysed and analysed by immunoblotting.

MKRN1 is a potential oncogene, based on its ability to degrade several tumour suppressors, including p53 and p21 (refs 22, 23, 24). In support of these observations, MKRN1 is overexpressed in human cervical and breast carcinomas^24^. To further define the oncogenic properties of MKRN1, the signalling pathways of various kinases in MKRN1-depleted HeLa cells were screened using dot blots of 34 antibodies against specific kinases. Intriguingly, MKRN1 depletion was associated with the suppression of pAKT, pmTOR and p-p70 S6K, which are critical components of PI3K/AKT-mediated oncogenic signalling pathways (Fig. 1a). Furthermore, a decrease in the levels of pAKT or its downstream phosphoproteins, including p70 S6K and GSK3β, was detected in both MKRN1^−/−^ mouse embryonic fibroblasts (MEFs) and HeLa cells depleted of MKRN1 by RNAi #6 or #7 (Fig. 1b,c). For controls, p53 and p14ARF as targets of MKRN1 are detected here (Fig. 1b,c)^23^,^25^. The *in vitro* phosphorylation analysis of AKT in the absence of MKRN 1 further confirms that MKRN1 depletion induces AKT inactivation (Fig. 1d). Because PTEN is the crucial antagonist of the PI3K/AKT proto-oncogenic axis, we also analysed PTEN levels in MKRN1-depleted MEFs or HeLa cells. MKRN1 ablation induced an increase in PTEN protein expression but did not have a substantial effect on PTEN messenger RNA levels, suggesting a potential role of MKRN1 in the post-translational regulation of PTEN (Fig. 1b,c,e). The simultaneous down- and upregulation of AKT and PTEN, respectively, by MKRN1 ablation led us to examine whether the inhibition of AKT signalling by MKRN1 elimination is dependent on an increase in PTEN. The ablation of PTEN completely reversed the effect of MKRN1 knockdown on pAKT expression and that of its downstream effectors (Fig. 1f). Compellingly, PTEN depletion induced the activation of AKT and also led to an increase in MKRN1, suggesting that active pAKT might affect MKRN1 (Fig. 1f, lane 4). Collectively, MKRN1 depletion appears to induce PTEN stabilization, consequently leading to the deactivation of AKT signalling pathways. By contrast, PTEN depletion led to the activation of AKT and an increase in MKRN1, suggesting an association among pAKT, MKRN1 and PTEN.

### MKRN1 overexpression correlates with tumour progression

**Figure 2:**
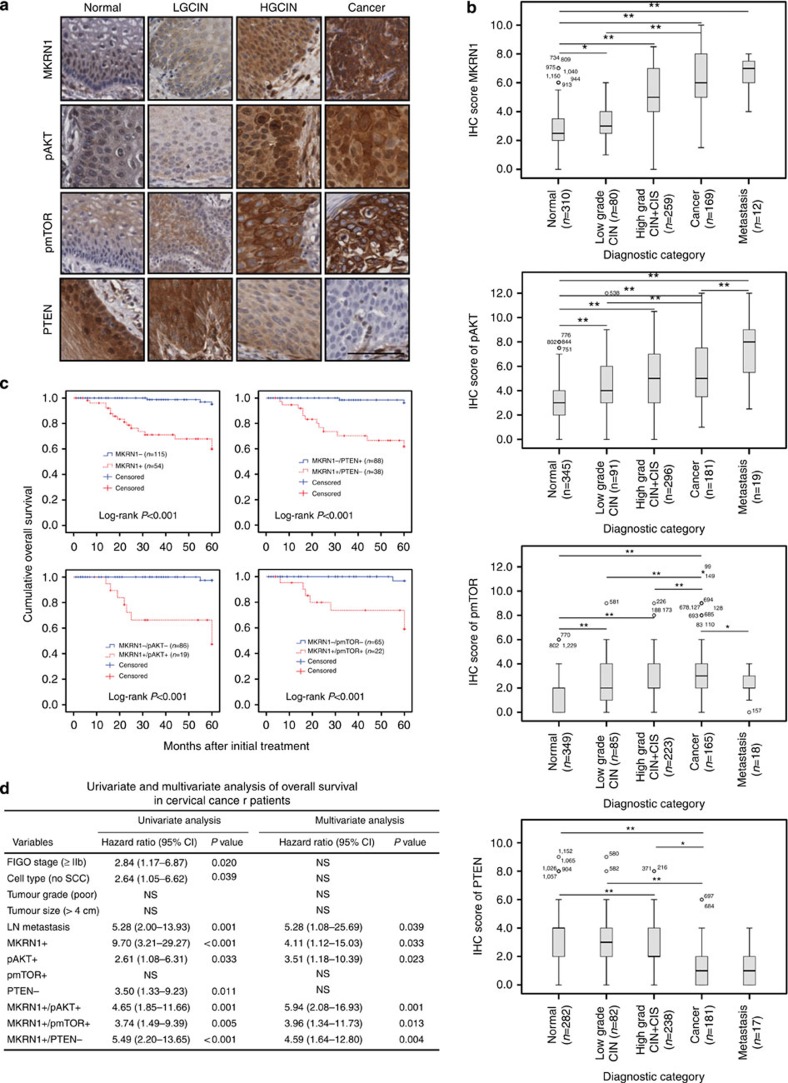
Determining protein expression in human cervical neoplasia specimens. The tissue microarray (TMA) contains 190 cases of cervical cancer. However, due to the complexity of sectioning and staining and sample heterogeneity, only 169, 181, 165 and 181 of the 190 samples could be interpreted for MKRN1, pAKT, pmTOR and PTEN expression, respectively. (**a**) Representative images of immunohistochemical staining of MKRN1, pAKT, pmTOR and PTEN in normal, low-grade CIN, high-grade CIN and invasive cervical cancer tissues. The boxed regions are displayed at high magnification in the inset. Scale bars, 200 μm. (**b**) Box plot depiction of IHC scores. IHC staining scores for MKRN1 and pAKT were significantly higher in cervical cancer samples than in low-grade CIN and normal controls. By contrast, the IHC score for PTEN was significantly lower in cancer samples than in CIN and normal controls. Error bars represent mean±s.d. IHC scores were compared by one-way ANOVA and an independent *t*-test. (*p<0.01 and **p<0.001). (**c**) Kaplan–Meier plots indicate the overall survival for cervical cancer patients categorized by tumour stage, LN metastasis, or MKRN1, pAKT, pmTOR or PTEN expression. (**d**) Univariate and multivariate analyses of the associations between prognostic variables and overall survival in cervical cancer. MKRN1+, histoscore ≥8; pAKT+, histoscore ≥8; pmTOR+, histoscore ≥3; PTEN-, histoscore <1; LN metastasis, lymph node metastasis. Data were analysed using a one-way ANOVA and an independent *t*-test.

On the basis of the results presented in Fig. 1, the clinical implications of AKT, MKRN1 and PTEN levels were pursued. First, to determine the clinical relevance of MKRN1 expression in human cancer, we used immunohistochemistry (IHC) to compare MKRN1 expression levels in human cervical tissue from patients with cervical intraepithelial neoplasia (CIN) or invasive cervical carcinoma. The clinicopathological characteristics of the study are summarized in Supplementary Table 1. The expression of MKRN1 was increased in CIN and tumours compared with normal cells (*P*<0.001, one-way analysis of variance (ANOVA) and independent *t*-test) (Fig. 2a,b; Supplementary Fig. 1). The expression of pAKT and pmTOR was also higher in CIN and tumours than in normal cells, whereas the expression of PTEN was decreased in CIN and tumour tissue compared with normal cells, suggesting a link between MKRN1 and the PI3K/AKT pathway (Fig. 2; Supplementary Table 2). Furthermore, MKRN1 immunoreactivity was significantly correlated with advanced disease and poor outcome, including the FIGO stage (*P*=0.018, one-way ANOVA and independent *t*-test) and tumour grade (*P*<0.001, one-way ANOVA and independent *t*-test). Similar results were obtained for pAKT expression (Fig. 2b; Supplementary Table 1). By contrast, the expression of PTEN was upregulated in normal specimens compared with cancer and high-grade CIN (*P*<0.001, one-way ANOVA and independent *t*-test, Fig. 2b). Notably, in all cervical cancer and CIN specimens, the expression of MKRN1 was positively correlated with the expression of pAKT (Spearman's rho=0.167 (*P*=0.030) and Spearman's rho=0.411 (*P*<0.001), respectively), whereas MKRN1 expression was negatively correlated with PTEN expression (Spearman's rho=−0.260 (*P*=0.001) and Spearman's rho=−0.175 (*P*=0.002), respectively, Supplementary Table 2). In addition, after further defined analysis in the CIN subgroups, the LGCIN group did not show statistically meaningful negative correlation (Spearman's rho=−0.020, *P*=0.861), HGCIN (Spearman's rho=−0.133, *P*=0.045) and Cancer (Spearman's rho=−0.175, *P*=0.002) group showed a strong negative correlation between MKRN1 and PTEN. The discrepancy between LGCIN and HGCIN/Cancer resulted from pathophysiological characteristics, because human papilloma virus (HPV) replication in LGCIN is without cell proliferation, whereas in HGCIN, cell proliferation occurs, resulting in the progression towards cervical cancer (Supplementary Table 2). We next examined the relationship between MKRN1 expression and patient survival. Kaplan–Meier plots demonstrated that patients with high MKRN1 expression had significantly worse overall survival (mean of 47.6 versus 59.5 months, log-rank test *P<*0.001; Fig. 2c). Furthermore, patients with a combination of high MKRN1 and high pAKT expression or high MKRN1 and low PTEN expression had significantly worse overall survival (mean of 46.5 versus 59.8 months, log-rank test *P<*0.001, and mean of 46.9 versus 59.5 months, log-rank test *P<*0.001, respectively) than patients who had low MKRN1 and low pAKT expression or low MKRN1 and high PTEN expression (Fig. 2c; Supplementary Fig. 2). The Cox proportional hazards model revealed that high MKRN1 expression (hazard ratio=4.11 (95% confidence interval (CI), 1.12–15.03), *P*=0.033), a combination of high MKRN1 and high pAKT (hazard ratio=5.94 (95% CI, 2.08–16.93), *P*=0.001) or high MKRN1 and low PTEN (hazard ratio=4.59 (95% CI, 1.64–12.80), *P*=0.004) expression were independent prognostic factors with respect to overall survival (Fig. 2d). Taken together, these data indicate that MKRN1 expression is an important prognostic factor in human cervical cancer, possibly due to its role in modulating a PTEN-dependent AKT inhibition pathway.

### AKT activation suppresses PTEN by MKRN1 stabilization

**Figure 3:**
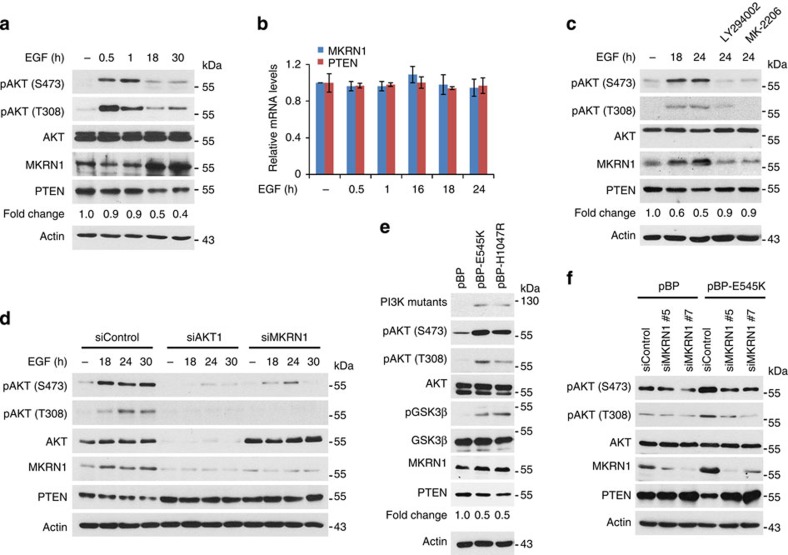
Active AKT induces the up- and downregulation of MKRN1 and PTEN. (**a,b**) EGF-dependent AKT activation stabilizes MKRN1 and destabilizes PTEN. ME-180 cells were serum starved (0.2% serum) for 24 h, treated with 100 ng ml^−1^ EGF for the indicated time and then analysed by immunoblotting (right panel). Total RNA was purified from EGF (100 ng ml^−1^)-stimulated ME-180 cells, and mRNA levels of MKRN1 and PTEN were measured using real-time PCR (left panel; data shown are mean±s.d.; *n*=3). (**c**) The effect of EGF treatment on the up- and downregulation of MKRN1 and PTEN, respectively, is PI3K/AKT dependent. After serum starvation, ME-180 cells were treated with EGF (100 ng ml^−1^) and LY294002 (PI3K inhibitor) or MK-2206 (1 μM, AKT inhibitor), as indicated. (**d**) EGF-dependent destabilization of PTEN is pAKT/MKRN1 axis dependent. ME-180 cells were transfected with AKT1 siRNA (siAKT1) or siMKRN1 #7, serum starved for 24 h after transfection and subsequently treated with EGF (100 ng ml^−1^) at the indicated time point. Cells were harvested at 72 h post transfection and analysed by immunoblotting. (**e,f**) The constitutively active PI3K mutants up- and downregulate MKRN1 and PTEN, respectively. Immunoblotting of lysates from ME-180 cells stably expressing PIK3CA p110α E545K or H1047R (**e**). ME-180 cells stably expressing PIK3CA p110α E545K were transfected with siMKRN1 #5 or siMKRN1 #7 and analysed by immunoblotting (**f**). (**a,c,e**) Relative PTEN protein expression levels are reported below the corresponding western blot bands.

The cellular and clinical data implicating potential associations among AKT, MKRN1 and PTEN led us to hypothesize that the PI3K/AKT signalling pathway might exert negative regulatory effects on PTEN stabilization, possibly via MKRN1. Mechanistically, the PI3K/AKT axis leading to PTEN deregulation is unknown, but it has been suggested that PIP3 participates in the regulation of PTEN ubiquitination and stability^26^,^27^. To unravel the possible connection among AKT, MKRN1 and PTEN, AKT was activated via epidermal growth factor (EGF) treatment following serum starvation of ME-180 cervical cancer cells. AKT immediately attained its activated pAKT state following EGF treatment, as previously reported (Fig. 3a). Intriguingly, MKRN1 levels increased at 18 h after EGF treatment, simultaneous with a decrease in PTEN protein expression. There was no apparent change in the messenger RNA level of either protein, suggesting that these events depend on post-translational processes (Fig. 3a,b). The MKRN1 stabilization and PTEN destabilization induced by EGF treatment were inhibited by either treatment with LY294002 (a PI3K inhibitor) and MK-2206 (an allosteric AKT inhibitor) or AKT knockdown (Fig. 3c,d). In contrast, treatment with PD98059 or AZD6244, MEK inhibitors, did not have much effect on EGF-mediated phosphorylation of pAKT, MKRN1 stabilization or PTEN destabilization, suggesting that the EGF–MAPK pathway might not be involved in these processes (Supplementary Fig. 3b). Note that MEK inhibitors suppressed EGF-induced ERK phosphorylation within 30 min. Furthermore, ERK pathway was inhibited at 8 h after EGF treatment by the negative-feedback loop previously reported (Supplementary Fig. 3a,b). These observations imply that EGF-driven PI3K/AKT activation might be related to the protein stabilities of MKRN1 and PTEN. Results similar to those shown for ME-180 were also observed in other cell lines, including H1299, HepG2 and Hep3B cells and MEFs, indicating that EGF-driven regulation of MKRN1 and PTEN is not only restricted in cervical cancer cell lines (Supplementary Fig. 3c). Furthermore, the inhibition of EGF-mediated PTEN destabilization upon MKRN1 ablation indicates that MKRN1 might have a regulatory effect on PTEN stabilization (Fig. 3d). To further elucidate the regulatory role of EGF in the interaction among AKT, MKRN1 and PTEN, the effects of PI3K on MKRN1 and PTEN levels were investigated. As previously reported, the stable overexpression of the constitutively active PI3K mutants PIK3CA p110α E545K or H1047R induced pAKT and pGSK3β, with simultaneous stabilization of MKRN1 and destabilization of PTEN (Fig. 3e)^28^,^29^. By contrast, MKRN1 ablation suppressed PI3K-dependent pAKT activation but stabilized PTEN (Fig. 3f). Collectively, these results suggest that the PI3K/AKT axis may induce concurrent post-translational stabilization of MKRN1 and destabilization of PTEN.

### AKT stabilizes MKRN1 through phosphorylation

**Figure 4:**
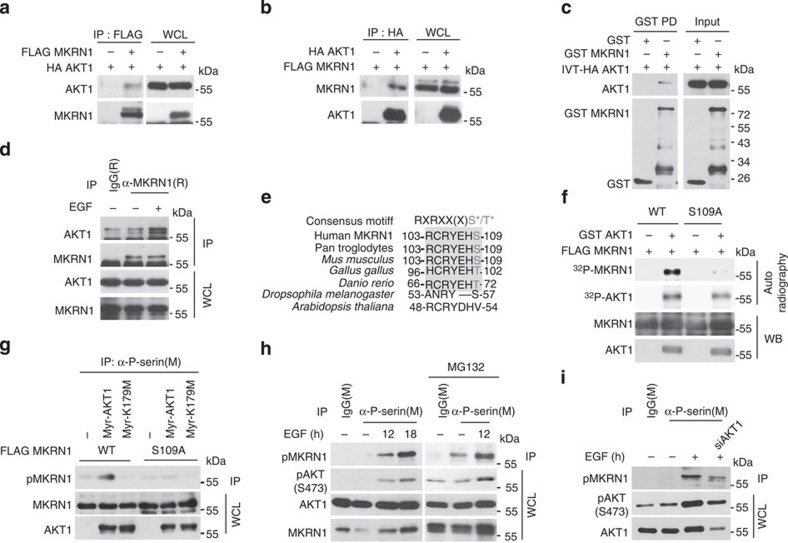
AKT phosphorylates serine 109 of MKRN1. (**a,b**) The interaction between ectopically expressed MKRN1 and AKT1 was demonstrated using a co-immunoprecipitation assay. (**c**) A GST pull-down assay revealed the direct interaction between MKRN1 and AKT1. GST-MKRN1 purified from bacteria and *in vitro* translated HA-AKT1 were incubated under cell-free conditions, and GST-MKRN1 was pulled down by glutathione Sepharose beads. (**d**) Endogenous MKRN1 was immunoprecipitated from ME-180 cells with or without EGF treatment (100 ng ml^−1^), and MKRN1-bound endogenous AKT1 was immunoblotted. (**e**) A consensus AKT phosphorylation site is present in MKRN1. (**f**) AKT directly phosphorylates MKRN1 WT but not the S109A mutant. An *in vitro* phosphorylation assay was performed using bacterially produced GST-AKT1 and FLAG-MKRN1 (WT or S109A), which was purified from FLAG-MKRN1-transfected HEK293T cells. Purified proteins were incubated with [γ-^32^P]ATP, and ^32^P incorporation was detected by autoradiography. (**g**) Constitutively active, but not inactive, AKT phosphorylates MKRN1 on serine 109. H1299 cells were co-transfected with FLAG-MKRN1 (WT or S109A) and HA-tagged Myr-AKT or K179M. Phosphorylation of ectopically expressed MKRN1 was detected by an *in vivo* phosphorylation assay (IP panel); WCL was also analysed. (**h**) AKT phosphorylates MKRN1 upon EGF treatment. After serum starvation, ME-180 cells were stimulated by EGF (100 ng ml^−1^) in the absence or presence of MG132 (10 μM) for 4 h and were examined in an *in vivo* phosphorylation assay. Endogenous phospho-MKRN1 was immunoprecipitated using an anti-phosphoserine antibody (IP panel), and WCL was immunoblotted. (**i**) EGF-induced MKRN1 phosphorylation is inhibited by AKT ablation. ME-180 cells were transduced with control siRNA or AKT1 siRNA (siAKT1) and subsequently serum starved, followed by treatment with EGF (100 ng ml^−1^). Cell extracts were analysed using an *in vivo* phosphorylation assay. Endogenous phospho-MKRN1 is indicated in the IP panel.

Given that EGF stabilizes the MKRN1 protein, possibly via activation of the PI3K/AKT axis, we investigated whether MKRN1 is a substrate for AKT. Employing co-immunoprecipitation assays using endogenous, overexpressed or recombinant proteins, an interaction between AKT1 and MKRN1 was detected (Fig. 4a–d). A search for a potential MKRN1 phosphorylation site revealed a consensus AKT phosphorylation motif including serine 109 of MKRN1 (Fig. 4e). Although the motif (RXRXXXS), including serine 109 of MKRN1, is only a partial AKT consensus motif (RXRXXS/T), an AKT substrate containing an RXRXXXS motif has been reported. Furthermore, previous structural studies have indicated that the RXRXXXS motif is a highly plausible AKT-targeting sequence^30^,^31^. To determine whether AKT targets the serine 109 site of MKRN1 for phosphorylation, we substituted alanine at this site. A subsequent *in vitro* phosphorylation assay using^32^ P-labelled adenosine triphosphate (ATP) and purified GST-AKT under cell-free conditions revealed that wild-type (WT) MKRN1 was phosphorylated, but not an S109A mutant (Fig. 4f). In accordance with this finding, only Myr-AKT (the active form of AKT) but not K179M (a kinase-dead form of AKT) was able to induce phosphorylation of WT MKRN1 but not the S109A mutant (Fig. 4g). Upon EGF treatment, an anti-phosphoserine antibody specifically detected pMKRN1, and MKRN1 was stabilized (Fig. 4h). Depletion or inhibition of AKT by short interfering RNA (siRNA) suppressed EGF-driven MKRN1 phosphorylation (Fig. 4i). Finally, phospho-AKT substrate antibody detecting phosphorylation on the overexpressed MKRN1 but not on the S109A mutant by Myr-AKT1 further suggests that the serine 109 site of MKRN1 is specifically phosphorylated by AKT (Supplementary Fig. 4). These data suggest that MKRN1 might be post-translationally stabilized via AKT-mediated phosphorylation.

**Figure 5:**
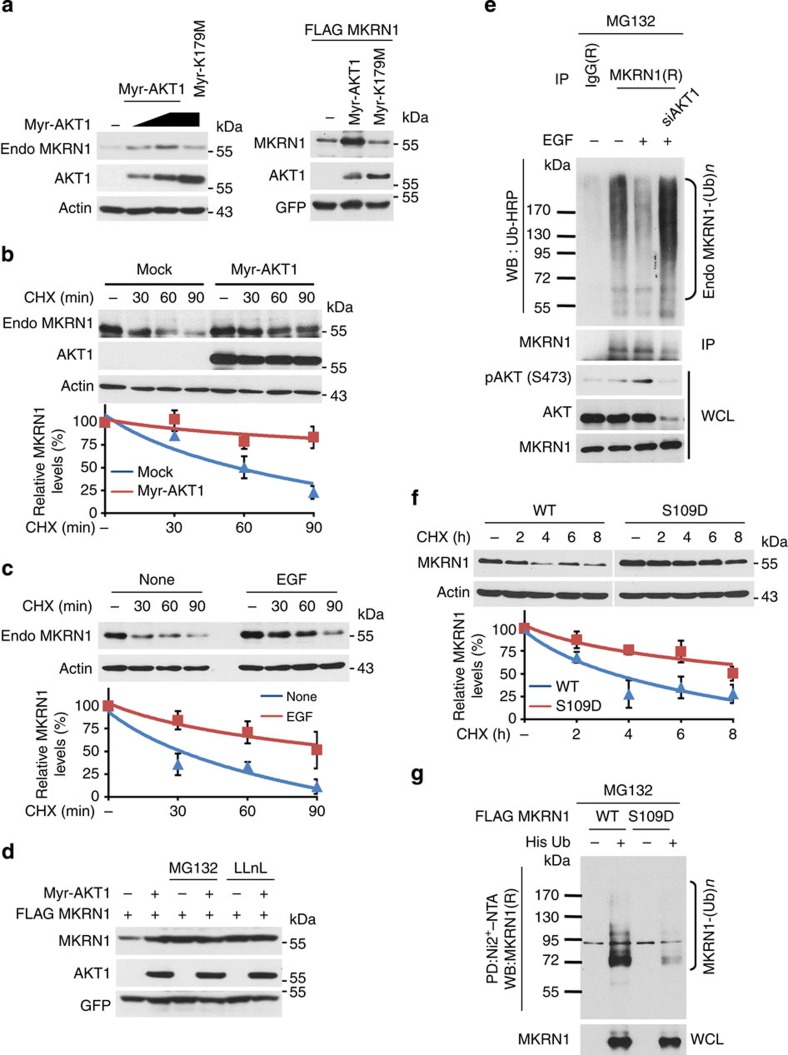
Active AKT induces the stabilization of MKRN1. (**a**) Overexpression of HA-tagged Myr-AKT but not K179M induces increased levels of endogenous MKRN1 or ectopically expressed FLAG-MKRN1 in H1299 cells. (**b**) Myr-AKT stabilizes endogenous MKRN1. H1299 cells were transfected with the indicated plasmid for 24 h and then treated with CHX (100 μg ml^−1^) at the indicated time points. (**c**) The half-life of the endogenous MKRN1 protein was determined in EGF (100 ng ml^−1^)-stimulated ME-180 cells. (**b,c**) The amount of MKRN1 was determined using western blotting after normalization to actin. (bottom panel, data shown are means±s.d.; *n*=3). (**d**) H1299 cells transfected with the indicated plasmid were treated with 10 μM MG132 or LLnL for 4 h. (**e**) EGF-dependent MKRN1 ubiquitination is reduced by AKT ablation. ME-180 cells transduced with siAKT1 were treated with EGF (100 ng ml^−1^), followed by MG132 (10 μM) for 4 h. The lysates were immunoprecipitated using an anti-MKRN1 antibody, followed by immunoblotting with an HRP-conjugated anti-Ub antibody under denaturing conditions. (**f**) The protein half-life of the S109D mutant is longer than that of the WT protein. H1299 cells were transfected with FLAG-MKRN1 WT or S109D and then treated with CHX (100 μg ml^−1^) for the indicated time points. Bottom panel: the graphs indicate the relative amounts of MKRN1 protein compared with the levels of actin in the western blot (data shown are means±s.d.; *n*=3). (**g**) Ubiquitination status of the S109D mutant. H1299 cells were transfected with the indicated plasmids and then treated with MG132 (10 μM). Cells were lysed in 6 M guanidine-HCl, and ubiquitinated proteins were purified using Ni^2+^-NTA beads. His-purified proteins were detected by immunoblotting.

To further demonstrate the link between the AKT-mediated phosphorylation and stabilization of MKRN1, the effects of constitutively active Myr-AKT or inactive AKT (K179M) on MKRN1 were analysed. As expected, Myr-AKT increased MKRN1 protein levels, while K179M did not (Fig. 5a). Cycloheximide (CHX) treatment indicated that Myr-AKT expression or EGF treatment prolongs the half-lives of endogenous MKRN1, confirming that active AKT stabilizes MKRN1 (Fig. 5b,c). Treatment of cells with the proteasome inhibitors MG132 and LLnL resulted in the full recovery of basal MKRN levels to that mediated by Myr-AKT, demonstrating that MKRN1 is constantly destabilized via the proteasomal degradation pathway (Fig. 5d). On the basis of these observations, the ubiquitinated forms of MKRN1 were measured in response to EGF treatment. The results indicated that the ubiquitinated MKRN1 forms disappeared upon EGF treatment (Fig. 5e; Supplementary Fig. 5). By contrast, the ablation of AKT restored ubiquitinated MKRN1, indicating that the inhibition of MKRN1 ubiquitination is dependent on EGF-mediated AKT activation (Fig. 5e). We next examined whether AKT-mediated MKRN1 phosphorylation affects the ubiquitination process, because phosphorylation stabilizes MKRN1 proteins. We generated a phosphorylated MKRN1 mimetic named S109D in which serine 109 was replaced with an aspartate to mimic the effects of phosphorylation on the charge of serine 109. As expected, the S109D mutant was fully protected against destabilization by CHX treatment and exhibited decreased levels of ubiquitination compared with WT (Fig. 5f,g). In summary, the PI3K/AKT-mediated phosphorylation of MKRN1 hinders its ubiquitination, ultimately stabilizing the MKRN1 protein.

### MKRN1 functions as a PTEN E3 ubiquitin ligase

**Figure 6:**
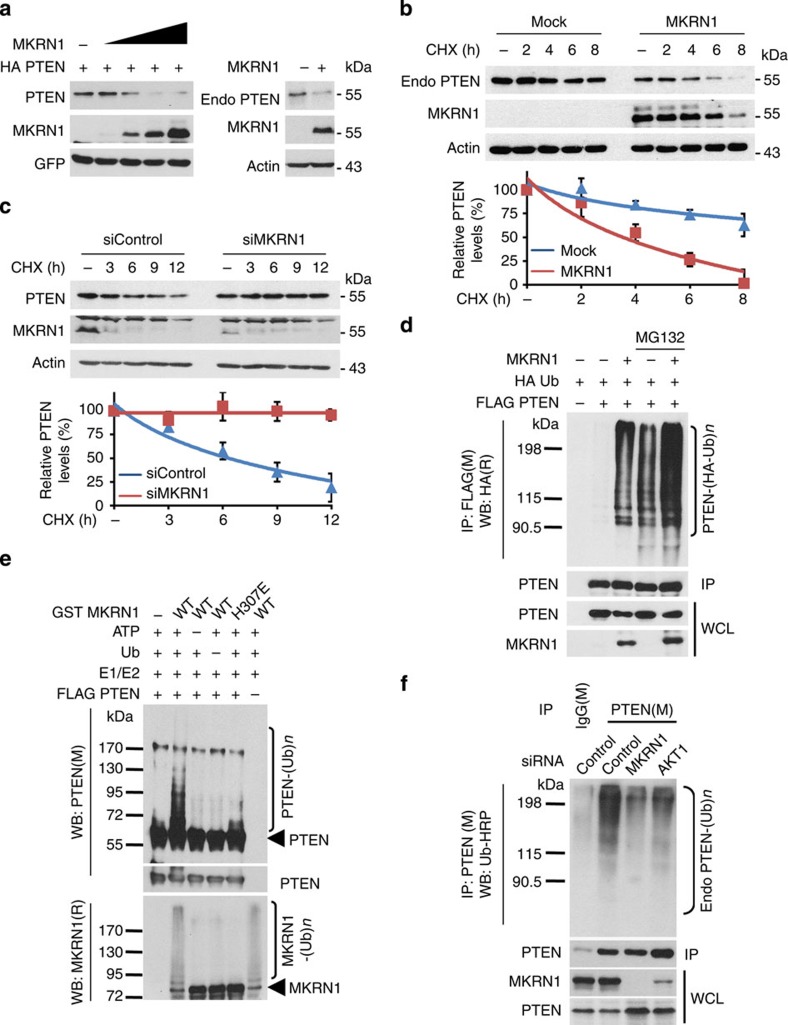
MKRN1 induces the ubiquitination and degradation of PTEN. (**a**) MKRN1 degrades both ectopically expressed PTEN and endogenous PTEN. H1299 cells were transfected with the indicated plasmid. GFP was used as the transfection control. (**b**) MKRN1 overexpression decreases endogenous PTEN stability. H1299 cells were transfected with the MKRN1 expressing plasmid for 24 h and then were treated with CHX (100 μg ml^−1^) at the indicated time points. (**c**) MKRN1 RNAi stabilizes PTEN. ME-180 cells were transduced with siControl or siMKRN1 #7, followed by the addition of CHX at the indicated time points. (**b,c**) The graph represents the values obtained after densitometry analysis. The percentage of the remaining PTEN protein after CHX addition is plotted (bottom panel, data shown are means±s.d.; *n*=3). (**d**) MKRN1 induces PTEN ubiquitination. To identify PTEN ubiquitination, H1299 cells were transfected with HA-Ub and the indicated plasmids, followed by treatment with MG132 (10 μM) for 6 h. HA-tagged ubiquitinated PTEN was purified by immunoprecipitation using an anti-FLAG antibody in 1% SDS buffer, followed by immunoblotting using an anti-HA antibody. (**e**) *In vitro* ubiquitination of PTEN via MKRN1. FLAG-PTEN proteins obtained from HEK293T cells using anti-FLAG beads were incubated with E1, E2 and ubiquitin (Ub) in the absence or presence of ATP along with GST, GST-MKRN1 or H307E (bacterially purified), as indicated for the *in vitro* ubiquitination of PTEN. (**f**) The ubiquitination status of endogenous PTEN upon MKRN1 or AKT1 ablation. Lysates from MKRN1- or AKT1-knockdown and MG132 (10 μM)-treated ME-180 cells were immunoprecipitated with an anti-PTEN antibody, and ubiquitinated PTEN was then immunoblotted using an HRP-conjugated anti-Ub antibody under denaturing conditions.

Our results provided several clues suggesting that MKRN1 is an E3 ubiquitin ligase for PTEN, including the results presented in Fig. 1. Figure 1 shows that MKRN1 depletion increased PTEN protein levels (Fig. 1b,c), while cells in which the PI3K/AKT/MKRN1 pathway was stimulated by EGF exhibited an MKRN1-dependent decrease in PTEN protein levels and (Fig. 3d). On the basis of these data, the potential role of MKRN1 as an E3 ligase for PTEN was investigated. First, co-immunoprecipitation analysis using overexpressed, endogenous and recombinant MKRN and PTEN revealed that MKRN1 binds to PTEN (Supplementary Fig. 6a–d). Domain mapping analysis suggested that the C terminus of MKRN1 is responsible for its interaction with PTEN (Supplementary Fig. 4e). The overexpression of MKRN1 in H1299 cells induced a decrease in endogenous, as well as exogenous, PTEN protein levels (Fig. 6a). The half-life of endogenous PTEN was also decreased by MKRN1 overexpression (Fig. 6b). On the other hand, the half-life of endogenous PTEN increased following MKRN1 depletion in cells treated with CHX (Fig. 6c). Ubiquitination assays performed under protein-denaturing conditions demonstrated that the ubiquitinated forms of exogenous PTEN were enhanced by the overexpression of MKRN1 but not the enzymatically defective H307E MKRN1 mutant (Fig. 6d; Supplementary Figs 7c and 8a). These findings support the data demonstrating that the overexpression of WT MKRN1, but not the H307E mutant, shortened the half-life of the exogenous PTEN protein (Supplementary Fig. 7a,b). *In vitro* ubiquitination assays conducted using either recombinant or cellular proteins demonstrated that MKRN1 directly facilitated the PTEN ubiquitination process (Fig. 6e; Supplementary Fig. 8b,c). To investigate the physiological implications of PTEN destabilization, the effect of MKRN1 or AKT depletion on the ubiquitination status of endogenous PTEN was examined. PTEN ubiquitination was sharply diminished by the ablation of either MKRN1 or AKT, suggesting that both MKRN1 and AKT are involved in PTEN ubiquitination processes (Fig. 6f). In addition, MKRN1 was shown to predominantly induce K48-linked polyubiquitination of PTEN (Supplementary Fig. 8d). Collectively, these analyses indicate that the stabilization of the MKRN1 E3 ligase by pAKT-mediated phosphorylation leads to PTEN ubiquitination and degradation, and that MKRN1 is a novel E3 ligase for PTEN.

### The active PI3K/AKT signal destabilizes PTEN

**Figure 7:**
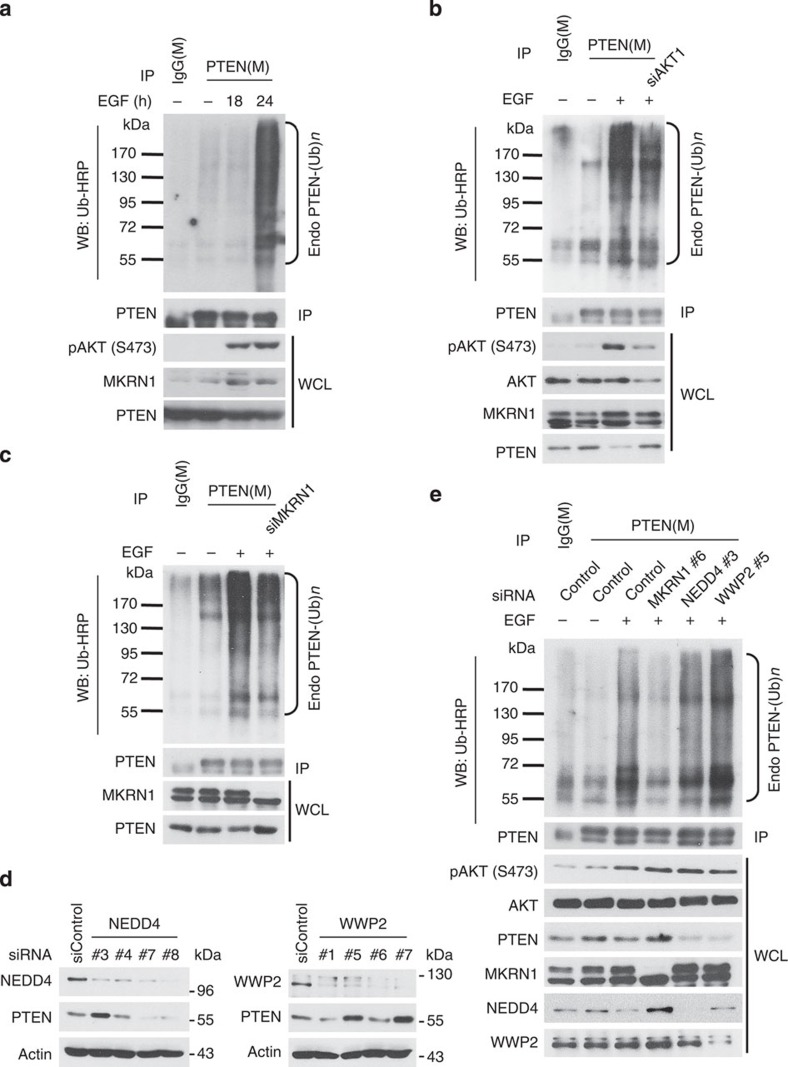
EGF induces PTEN ubiquitination in an AKT/MKRN1-dependent manner. (**a**) Endogenous PTEN ubiquitination is accelerated upon EGF treatment. ME-180 cells treated with EGF for the indicated times were lysed and immunoblotted with an anti-PTEN antibody in 1% SDS buffer, followed by immunoblotting with an HRP-conjugated anti-Ub antibody. (**b**) EGF-induced PTEN ubiquitination is blocked by AKT ablation. ME-180 cells were transduced with control siRNA or siAKT1 and then treated with EGF (100 ng ml^−1^) for 24 h. Ubiquitinated PTEN was immunoprecipitated and immunoblotted as described above. (**c**) EGF-induced PTEN ubiquitination was blocked by MKRN1 ablation. ME-180 cells transduced with siMKRN1 #7 were stimulated by EGF (100 ng ml^−1^) for 24 h. To validate endogenous PTEN ubiquitination, an *in vivo* ubiquitination assay was performed as described above. (**d**) Knockdown of NEDD4-1 or WWP2 in ME-180 cells. Immunoblotting of NEDD4-1 or WWP2 and PTEN in four types of NEDD4-1 siRNA- or WWP2 siRNA-transfected cells. (**e**) MKRN1, but not NEDD4-1, or WWP2 ablation suppresses EGF-dependent PTEN ubiquitination. ME-180 cells were transfected with control siRNA, siMKRN1 #7, NEDD4-1 #3 or WWP2 #5 as indicated and subsequently stimulated with EGF for 24 h. Endogenous PTEN ubiquitination was analysed by an *in vivo* ubiquitination assay.

The ability of the EGF/PI3K/AKT axis to activate MKRN1, which might consequently induce PTEN destabilization, led us to investigate whether this signalling pathway could affect the ubiquitination status of PTEN. The ubiquitination of endogenous PTEN was considerably increased 24 h after EGF treatment, with simultaneous activation of the pAKT/MKRN1 axis (Fig. 7a). Notably, PTEN ubiquitination decreased upon AKT or MKRN1 ablation, indicating that EGF-driven PTEN ubiquitination is pAKT- and MKRN1 dependent (Fig. 7b,c). Consistent with these observations, overexpression of Myr-AKT induced the endogenously ubiquitinated PTEN, which was suppressed in the absence of MKRN1 (Supplementary Fig. 9). The ubiquitination and subsequent degradation of PTEN are mediated by several E3 ligases, including NEDD4-1 and WWP2 (refs 16, 17). Thus, we examined whether other PTEN E3 ligases were involved in PTEN regulation similar to MKRN1. Consistent with the findings of previous studies, knockdown of either NEDD4-1 or WWP2 induced PTEN stabilization in ME-180 cells (Fig. 7d). siNEDD4-1 #3 and siWWP2 #5, which increased PTEN levels, were then used to determine whether NEDD and WWP2 are involved in PTEN regulation through EGF-mediated signalling pathways. Upon EGF exposure, only MKRN1 depletion fully interfered with the active EGF/PI3K/AKT axis-mediated ubiquitination of PTEN, whereas neither NEDD4-1 nor WWP2 had this effect (Fig. 7e). In addition, the known ubiquitination sites, K13 and K289, on PTEN by NEDD4 were tested for MKRN1's targeting. The results showed that MKRN1 could specifically target K289 but not K13 for ubiquitination. Supporting these data, MKRN1 could not mediate the degradation of K289R, implying that MKRN1 and NEDD4 have a common target site on PTEN (Supplementary Fig. 8e,f). Taken together, these results indicate that the EGFR/PI3K/AKT signal suppresses PTEN stability and activity through MKRN1-mediated ubiquitination and degradation.

### MKRN1 regulates the tumorigenicity of cervical cancer

**Figure 8:**
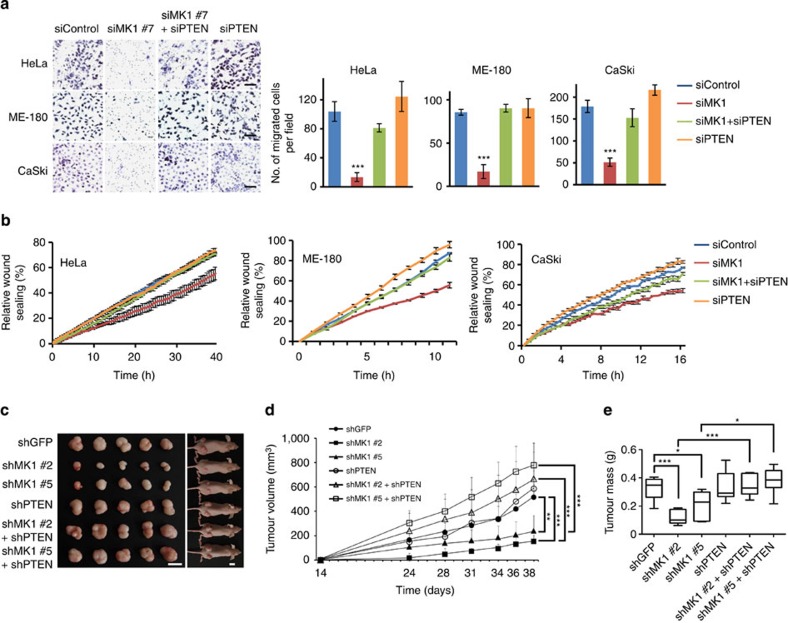
Loss of MKRN1 suppresses cervical cancer cell mobility and growth. (**a**) The migratory properties of siMKRN1 #7- and siPTEN-transfected cervical cancer cell lines (HeLa, ME-180 and CaSki) were analysed by a migration assay using a collagen-coated Transwell chamber. The photographic images represent haematoxylin and eosin-stained migratory cells (left panel, magnification: × 200, scale bar, 50 μm). Migratory cells were plotted as the average number of cells per field of view in three different experiments (right panel, data are shown as the mean±s.d. ***P<0.001, based on the Student's *t*-test.). (**b**) The scratch assay was performed using siMKRN1 #7- and siPTEN-transduced cervical cancer cell lines (HeLa, ME-180 and CaSki). The wound-healing images were analysed every 1 h or 30 min using the IncuCyte live-cell imaging system; the graph shows the percentages of sealing confluence as defined by the IncuCyte software (error bars represent mean±s.d.; *n*=3). (**c**–**e**) Representative images of tumours and mice at day 38 after the subcutaneous injection of 2 × 10^6^ ME-180 cells stably expressing shGFP or MKRN1 shRNA (shMK1 #2 or shMK1 #5) or PTEN shRNA (shPTEN) (**c**). Bars, 1 cm. Tumour growth is shown in the graph (**d**), and tumour mass is described at day 38 after implantation (**e**). The data shown in **d** and **e** are the mean±s.d.; *n*=6 mice per group in **c**–**e**. *P<0.05, **P<0.01 and ***P<0.001, based on the Student's *t*-test.

Several studies have demonstrated that PTEN-mediated tumour growth and inhibition of metastasis are related to the suppression of the PI3K/AKT pathway^5^. To determine the oncogenic effects of MKRN1-mediated inhibition of the PTEN tumour suppressor and activation of the PI3K/AKT pathway, we conducted a transwell migration assay using human cervical cancer cell lines (HeLa, ME-180 and CaSki). The migration abilities of MKRN1-depleted cells were strongly reduced (Supplementary Fig. 10, left panel). The number of migratory cells was reduced to <20% that in the control upon treatment with the individual MKRN1 RNAis (Supplementary Fig. 10, right panel). As expected, the inhibition of cell migration by MKRN1 depletion was reversed in PTEN-ablated cells (Fig. 8a). To confirm the effects of MKRN1 on cancer cell proliferation and motility, additional wound-healing assays were performed. To obtain live wound-healing images, RNA interference (RNAi)-transfected cells were injured, and IncuCyte was applied for further detection and analyses. Consistent with the transwell migration data, MKRN1-depleted cells exhibited an apparent reduction in migration rate compared with controls. This reduction was rescued by simultaneous PTEN ablation (Fig. 8b).

To investigate the inverse correlation between MKRN1 and PTEN in cervical cancer cell lines, *in vivo* xenograft analyses were performed. ME-180 cells from which MKRN1 had been stably eliminated were subcutaneously transplanted into athymic nude mice (Fig. 8c). The injected tumours that contained a retroviral-based MKRN1 short hairpin RNA (shRNA) vector exhibited distinct growth retardation compared with control shRNA vector-infected tumours. Notably, the concomitant stable depletion of PTEN completely reversed the tumour growth inhibition achieved by MKRN1 knockdown (Fig. 8c–e). The same phenomena were observed with cell lines tested *in vitro* (Supplementary Fig. 11a). Examination of the AKT signal in the tumour lysates revealed that MKRN1 knockdown diminished pAKT in a PTEN-dependent manner, in agreement with our previous observations (Supplementary Fig. 11b). Because MKRN1 could also target p53 or p14ARF, we further tested whether these factors might have some effects in the reverse correlation between MKRN1 and PTEN. For these, we employed HCT116 and ME-180, a colorectal and cervical cancer cell line, respectively. By using HCT116 p53^−/−^, we could remove p53 as well as p14ARF, which is ectopically suppressed in HCT116 cells^32^. Because ME-180 contains E6, which suppresses p53, stable knockdown of p14ARF enabled us to exclude the effects of both proteins. In these cells, the suppression of MKRN1 induced growth retardation, which was reversed by the knockdown of PTEN (Supplementary Fig. 12). Furthermore, using ME-180 stably transfected with ARF shRNA, we performed xenograft analysis in the absence of MKRN1 or MKRN1 and PTEN. The data showed that the deleterious effects of MKRN1 depletion on cells could be rescued by PTEN depletion alone in the xenografted tumours derived from the cervical cancer cell line (Supplementary Fig. 13). Notably, while the depletion of p53 relieved some of the tumour-suppressive effects upon MKRN1 ablation (Supplementary Fig. 12a), HCT116 still required depletion of PTEN for full growth recovery of cancer cells. Taken together, these results suggest that the loss of MKRN1 inhibits cervical cancer tumorigenesis through active PTEN stabilization.

## Discussion

PTEN is a major tumour suppressor that antagonizes growth factor-stimulated PI3K/AKT signalling by converting PIP3 to PIP2. PTEN loss of function is linked to various sporadic human cancers, including endometrial, glioblastoma, melanoma, lung, breast and prostate, with an average mutational frequency rate of ∼25% (refs 15, 33, 34). The frequent loss of heterozygosity in human cancers, the inverse correlation between PTEN dosage and tumorigenicity in a mouse model and the variety of PTEN regulatory mechanisms, including microRNA targeting and protein stability, suggest that variations in PTEN levels in cells might affect tumour progression^5^,^12^,^34^. Among those findings, the recent identification of the post-translational regulation of PTEN by phosphorylation, oxidation, acetylation, ubiquitination/deubiquitination and so on has generated widespread speculation on the implications of PTEN protein stability in tumorigenic processes^7^,^12^,^33^. While biochemical studies have investigated the post-translational regulation of PTEN associated with clinical behaviour, the detailed regulatory mechanisms of PTEN in a physiological context are not yet understood.

In this study, we identified a novel negative regulatory mechanism of PTEN involving EGF-dependent PI3K/AKT activation. Because PTEN's potent tumour-suppressing activities largely stem from its inhibitory effects on AKT activation, cells, whether normal or cancerous, must find ways to suppress PTEN during the active stimulation of cellular proliferation by cell growth signalling. Evidence indicating that growth factor signalling pathways post-translationally eliminate the inhibitory effects of PTEN has been limited. The increase in PTEN ubiquitination upon EGF stimulation but not in the absence of AKT or MKRN1 suggests the presence of coordinated pathways between the EGFR/AKT/MKRN axis and PTEN (Fig. 7). In fact, EGF-stimulated pAKT phosphorylates and subsequently stabilizes MKRN1, which then ubiquitinates and induces the degradation of PTEN. The stabilization of MKRN1 by EGF-mediated AKT activation with concomitant PTEN degradation suggests that a positive-feedback pathway activates AKT (Fig. 1f; Fig. 3). MKRN1 appears to be the only E3 ligase associated with the EGF-activated AKT pathway (Fig. 7).

Although persistent infection with high-risk HPV is a major factor for the development of cervical carcinomas, high-risk HPV alone is not sufficient to induce tumour progression^35^. Less than 4% of HPV-infected individuals acquire premalignant lesions or develop tumours, suggesting a role for host factors associated with HPV^11^,^36^. Recent genome-wide association studies (GWAS) and DNA sequencing data on human cervical cancer have revealed that candidate host factors with somatic mutations include MAPK1, RAS, ERBB2, PTEN and PIK3CA^11^,^37^. Among these proteins, mutations in ERBB2, RAS and PIK3CA may result in the constitutive activation of AKT. Active AKT could then stimulate its downstream effectors, such as MKRN1, leading to the suppression of PTEN; this mechanism may be reflected in the IHC and survival data in Fig. 2. While the above-indicated relationships between AKT and its downstream factors require further analyses in cervical cancer patients, our observations are sufficiently consistent with GWAS of cervical carcinomas to postulate that PTMs of AKT, MKRN1 and PTEN play important roles in coordinating the development of malignant cancers. Furthermore, the detection of a non-functional PTEN mutant in GWAS of cervical cancers implies that the impairment of PTEN-related pathways might be involved in the development of cervical cancer^11^. Our novel findings regarding the EGF/PI3K/AKT/MKRN1 axis leading to PTEN suppression, along with previous data on mutations in ERBB2, and PIK3CA, ultimately indicate that ERBB2- or PIK3CA-targeted drugs might be potential therapeutic treatments for cervical cancers.

## Methods

### Cell culture and transfection

HeLa, ME-180 and CaSki cells (human cervical cancer cell lines) were procured from the Korean Cell Line Bank (KCLB, Seoul, Korea) and cultured in RPMI supplemented with 10% fetal bovine serum and 1% penicillin/streptomycin (Thermo Scientific). H1299 (a human lung carcinoma cell line), HEK293T (a human embryonic kidney cell line) and MKRN1^+/+^ or MKRN1^−/−^ MEFs^25^ were cultured in DMEM with 10% fetal bovine serum and 1% penicillin/streptomycin (Thermo Scientific). H1299 and HEK293T were purchased from the American Type Culture Collection (ATCC, Manassas, VA). The ATCC and KCLB authenticate the phenotypes of these cell lines on a regular basis. All cell lines were maintained in 5% CO_2_ at 37 °C. Plasmid DNA was transfected using Lipofectamine 2000 (Invitrogen, Carlsbad, CA, USA), and siRNA was transfected using Lipofectamine RNAiMAX (Invitrogen) according to the manufacturer's protocol. For EGF stimulation, cells were starved overnight in medium supplemented with 0.2% serum, followed by treatment with EGF (100 ng ml^−1^; Sigma-Aldrich, St Louis, MO).

### Plasmids

The MKRN1 open-reading frame (ORF)-containing plasmids pcDNA3.1-MKRN1 WT/H307E, pcDNA3-HA-MKRN1 WT/H307E, pcDNA3-FLAG-MKRN1 WT/H307E and pGEX-4T-1-GST-MKRN1 WT/H307E were described previously^23^. MKRN1 S109A/S109D plasmids were generated by site-directed mutagenesis and subcloned into a pcDNA3-FLAG vector. The full-length PTEN ORF was kindly provided by Han-Woong Lee (Yonsei University) and subcloned into pcDNA3-HA or pcDNA3-FLAG. The AKT1 ORF-containing plasmids pLNCX-Myr-HA-Akt1-WT and K179M were purchased from Addgene (Cambridge, MA, USA), and the AKT1 ORF was subcloned into pcDNA3.1. The PIK3CA ORF-containing plasmids pBabe-puro-HA-PIK3CA E545K and H1047R were purchased from Addgene. pcDNA3-His-Ub was kindly provided by D.P. Lane^38^. pHM6-HA-Ub has been described previously^39^. pEGFP-C2 (Clontech, San Diego, CA) was used as a transfection control.

### Antibodies and chemicals

The following antibodies were used: PTEN (1:1,000, Santa Cruz, SC-7974 mouse, clone A2B1; or 1:1,000, Cell Signaling, 9559 rabbit), MKRN1 (1:1,000, Bethyl Laboratories, A300-990A), NEDD4-1 (1:1,000, Cell Signaling, 3607), WWP2 (1:1,000, Bethyl Laboratories, A302-936A), pAKT (Ser473, 1:2,000, Cell Signaling, 9271; T308, 1:2,000, Cell Signaling, 4056), AKT1 (1:2,000, Cell Signaling, 2967 mouse, clone 2H10), AKT1/2 (1:2,000, Santa Cruz, SC-8312 rabbit), p-p70 S6 kinase (Thr389, 1:2,000, Cell Signaling, 9205), p70 S6 kinase (1:2,000, Cell Signaling, 2708), pGSK3β (Ser9, 1:3,000, Cell Signaling, 9323), GSK3β (1:3,000, Cell Signaling, 9315), pERK1/2 (Thr202/Tyr204, 1:3,000, Cell Signaling, 4370), ERK1/2 (1:3,000, Cell Signaling, 4695), phosho-AKT substrate (RXRXXpS/pT, Cell Signaling, 10001), horseradish peroxidase (HRP)-conjugated-α-Ub FK2 (1:2,000, Biomol, PW0150), phosphoserine (Sigma, P5747), haemagglutinin (HA) (1:1,000, Santa Cruz, sc-7392 mouse; 1:1,000, Santa Cruz, sc-805 rabbit; or 1:2,000, Roche, 12013819001, clone 3F10), FLAG (1:5,000, Sigma, F3165 mouse; or 1:2,000, Sigma, F7425 rabbit), GST (1:1,000, Santa Cruz, sc-138), green fluorescent protein (GFP) (1:2000, Santa Cruz, sc-8334) and β-actin (1:5,000, Sigma, A5316). Human EGF (E9644), LY294002 (L9908), LLnL (A6185) and CHX (C4859) were purchased from Sigma-Aldrich. PD98059 (153000) and MG132 were purchased from Calbiochem (San Diego, CA). MK-2206 (S1078) and AZD6244 (S1008) were purchased from Selleck Chemicals.

### Protein assays

WT and H307E GST-MKRN1 were purified from bacteria using GST Sepharose beads according to the manufacturer's protocol (GE Healthcare). Recombinant GST-AKT1 protein (Biomol International, SE-416) and recombinant His-PTEN (Calbiochem, 481409) were purchased. *In vitro* translated IVT-HA-AKT1 and IVT-FLAG-PTEN proteins were obtained using a TNT T7-coupled reticulocyte lysate system (Promega, L4610). FLAG-MKRN1 WT/S109A and FLAG-PTEN proteins were purified using ANTI-FLAG M2 Affinity Gel (Sigma, A2220) and FLAG peptide (Sigma, F3290) from HEK293T cells expressing FLAG-tagged proteins. To confirm their interaction *in vitro*, GST-tagged proteins were incubated with *in vitro* translated proteins for 2 h, followed by the addition of GST Sepharose beads and incubation for 1 h. Finally, the beads were washed and eluted in 10 mM reduced glutathione. To perform the immunoprecipitation assay, cells were lysed in lysis buffer (50 mM Tris-HCl (pH 7.5), 150 mM NaCl, 0.5% Triton X-100 and 1 mM EDTA) containing a protease inhibitor cocktail. The cell lysates were then incubated with 1 μg of antibody for 2 h with gentle rotation, followed by incubation with 25 μl of protein G agarose (Invitrogen) for 2 h. The beads were washed three times with lysis buffer free of protease inhibitors, and the precipitated proteins were eluted in SDS sample buffer under boiling conditions. Full blots are shown in Supplementary Fig. 14

### *In vivo* and *in vitro* ubiquitination assay

The *in vivo* ubiquitination assay was conducted under denaturing conditions. Briefly, to detect proteins ubiquitinated by His-conjugated ubiquitin, cells were lysed in 6 M guanidinium-HCl buffer (pH 8) containing 5 mM *N*-ethylmaleimide (NEM, Sigma-Aldrich) to prevent deubiquitination. Using Ni^2+^-NTA beads (Qiagen, Valencia, CA), His-ubiquitin-conjugated proteins were pulled down and washed. To detect proteins ubiquitinated with HA-conjugated-ubiquitin or endogenously ubiquitinated proteins under denaturing conditions, cells were lysed by boiling for 10 min in PBS containing 1% SDS and 5 mM NEM. The lysates were immunoprecipitated in lysis buffer (a final concentration of 0.1% SDS). For immunoblotting, proteins were transferred to polyvinylidene difluoride membranes and denatured using 6 M guanidine-HCl containing 20 mM Tris-HCl (pH 7.5), 5 mM mercaptoethanol and 1 mM phenylmethyl sulphonyl fluoride for 30 min at 4 °C. Ubiquitinated proteins were identified by HRP-conjugated anti-Ub antibodies (FK2, PW0150, Biomol). *In vitro* ubiquitination assays were performed by combining 0.5 μg of the bacterially produced recombinant proteins (GST-MKRN1 or His-PTEN) or 10 μl of the purified proteins (FLAG-MKRN1 or FLAG-PTEN) from HEK293T cells with 100 ng of E1 (UBE1, E-305, Boston Biochem, Cambridge, MA, USA), 250 ng of E2 (UbcH5c, E2-627, Boston Biochem) and 5 μg of ubiquitin (U-100H, Boston Biochem) in 20 μl of reaction buffer (40 mM Tris, 50 mM NaCl, 5 mM MgCl2, 2 mM ATP, 1 mM dithiothreitol, pH 7.6) as indicated. The reaction was stopped after 3 h at 37 °C by the addition of SDS sample buffer and boiling.

### Virus production and infection

Stable PIK3CA-expressing cell lines were developed using retroviral expression vectors (pBabe-puro-HA-PIK3CA H1047R or pBabe-puro-HA-PIK3CA E545K). HEK293T cells were transfected with pBABE-puro together with VSV-G and a gag-pol-expressing vector followed by incubation for 48 h to produce packaged retroviruses. Finally, to select for retrovirus-infected cells, cell lines were incubated in 2 μg ml^−1^ puromycin (Sigma-Aldrich). Lentivirus-containing supernatant was collected 48 h after co-transfection of the pLKO.1 shRNA-expressing vector and packaging vectors into HEK293T cells, and added to ME-180 cells. After 48 h, infected cells were selected by treatment with 2 μg ml^−1^ puromycin. The following MISSION lentiviral shRNA expression vectors for human MKRN1, human PTEN and control GFP were purchased from Sigma-Aldrich: shMKRN1 #2 (TRCN0000041205, 5′-CCGGCCAGAGGTCACAGCACATAAACTCGAGTTTATGTGCTGTGACCTCTGGTTTTTG-3′), shMKRN1 #5 (TRCN0000 324883, 5′-CGGGTGTTGGATCACTTGCTGAAACTCGAGTTTCAGCAAGTGATCCAACACTTTTTG-3′) and shPTEN #5 (TRCN0000230370, 5′-CCGG CCACAAATGA AGGGATATAAACTCGAGTTTATATCCCTTCATTTGTGGTTTTTG-3′).

### *In vivo* and *in vitro* phosphorylation assays

To detect ectopically expressed or endogenous phosphorylated MKRN1, EGF-stimulated cells were lysed in lysis buffer containing a 1:100 dilution of phosphatase inhibitor cocktail (p5726, Sigma-Aldrich). The lysates were immunoprecipitated overnight with 1 μg of anti-phosphoserine; 25 μl of protein G agarose beads was then added and incubated for an additional 2 h. The beads were washed three times with lysis buffer, and the precipitated proteins were eluted in SDS sample buffer under boiling conditions. Phosphorylated MKRN1 was detected by immunoblotting with an anti-MKRN1 antibody. To detect phosphorylated MKRN1 *in vitro*, FLAG-MKRN1 WT/S109A proteins (10 μl) were purified from HEK293T cells that were serum starved (0.2% serum) for 24 h and incubated with recombinant active GST-AKT1 protein (1 μg, Biomol International, SE-416) and 2 μCi of [γ-^32^P]ATP in 50 μl of 1 × kinase buffer (25 mM Tris-HCl pH 7.5, 5 mM β-glycerophosphate, 2 mM dithiothreitol, 0.1 mM Na_3_VO_4_, 10 mM MgCl_2_ and 2 μM unlabelled ATP) for 1 h at 30 °C. Reactions were stopped by boiling in SDS sample buffer, and proteins were resolved by 8% SDS–polyacrylamide gel electrophoresis. ^32^P incorporation was detected by autoradiography.

### siRNA sequences

All siRNAs were obtained from Qiagen and were resynthesized: MKRN1 #6 (5′-(CG)GGATCCTCTCCAACTGCAA-3′), MKRN1 #7 (5′-(CA)CAGGCGAAGCTGAGTCAAG-3′), PTEN (5′-ATCGATAGCATTTGCAGTATA-3′), AKT1 (5′-(AA)TCACACCACCTGACCAAGA-3′), NEDD4-1 (5′-(AG)CCTACAATCTCTTATTAAA-3′) and WWP2 (5′-(AA)GCGGATGCTCAATAAGAGA-3′).

### Reverse transcription–PCR analysis

Total RNA was prepared using TRIzol reagent (Invitrogen), and complementary DNA was amplified using 1 μg of total RNA and analysed using the QuantiTect SYBR Green PCR Kit and real-time PCR (Rotor-GeneQ 2plex, Qiagen) with custom Primetime qPCR Primers (IDT, Coralville, IA, USA): human MKRN1, 5′-GAGAAGGACATGGAGCTCTCA-3′ (forward) and 5′-CGCCTTGTTGCTCATTGCCTC-3′ (reverse); human PTEN, 5′-GATGAGGCATTATCCTGTACACA-3′ (forward) and 5′-CTCTTCAGATACTCTTGTGCTGT-3′ (reverse); and human glyceraldehyde-3-phosphate dehydrogenase, 5′-TGTAGTTGAGGTCAATGAAGGG-3′ (forward) and 5′-ACATCGCTCAGACACCATG-3′ (reverse).

### Human tumour samples

Cervical cancer tissue microarrays constructed by the Korea Gynecologic Cancer Bank included 190 cervical cancers, 316 high-grade CINs and 95 low-grade CINs. Primary tumour specimens were collected from 1996 to 2010. Patient consent was obtained. This study was approved by the institutional review board of the Gangnam Severance Hospital, Yonsei University College of Medicine (Supplementary Table 1).

### Immunohistochemistry and scoring

Immunohistochemical staining was performed as described previously^40^. MKRN1, pAKT, pmTOR and PTEN staining results were scored based on (a) intensity (categorized as 0 (absent), 1 (weak), 2 (moderate) or 3 (strong)) and (b) the percentage of positively stained epithelial cells (scored as 0 (0% positive), 1 (1–25%), 2 (26–50%), 3 (51–75%) or 4 (>75%)). A histoscore was generated by multiplying the intensity and positivity scores (overall score range, 0–12). The immunohistochemical cutoff for high expression of tumour markers was determined through receiver-operating characteristic curve analysis^41^. Slides were scored without knowledge of any clinical information, and the final staining score was the average of scores from two independent pathologists.

### Human phosphokinase array

Diluted lysates from siControl or siMKRN1 #7-transfected HeLa cells were incubated with human phosphokinase array membranes (R&D Systems, ARY003), and bound phosphoproteins were analysed according to the kit instructions. Each membrane contained kinase-specific and positive control antibodies spotted in duplicate. The relative phosphorylation of each spot was quantified by normalizing the pixel density to that of each positive control. Values were expressed as the mean intensity of the siMKRN1 #7 membrane relative to the mean intensity of the siControl membrane.

### Transwell migration and scratch wound healing

For the transwell migration experiment, human cervical cancer cell lines (HeLa, ME-180 and CaSki) were transfected with siMKRN1 #7 and siPTEN for 24 h, trypsinized, resuspended in serum-free medium and added to the upper chambers of pore inserts of a transwell with collagen type l (BD Bioscience)-coated filters. RPMI with 10% FBS and 1% antibiotics (Thermo Scientific) were added to the lower chamber and incubated for 24 h. Migrating cells were quantified after haematoxylin and eosin staining. Each experiment was performed in triplicate.

Human cervical cancer cell lines (HeLa, ME-180 and CaSki) transduced with siMKRN1 #7 and siPTEN were plated and incubated for 24 h, until confluence. A wound was scratched across each well (Wound Maker, Essen BioScience, MI, USA), and the wells were then rinsed with fresh medium to remove floating cells. The wound-healing process was continuously monitored using the IncuCyte live-cell imaging system (Essen BioScience). Wound healing was determined as a percentage of wound confluence.

### *In vivo* tumorigenesis study

All animal studies involving the use of nude mice were approved by the Animal Care and Use Committee of Yonsei University Medical School (2013-0339), and were performed in specific pathogen-free facilities in accordance with the Guidelines for the Care and Use of Laboratory Animals of YUMS. ME-180 cells (2 × 10^6^) stably expressing shRNA in 100 μl PBS (mixed with Matrigel (BD Sciences) at a 1:1 ratio) were injected subcutaneously into the flanks of 6-week-old female-specific pathogen-free Balb/c nude mice (Central Lab, Animal Inc., Korea) under anaesthesia. After the formation of palpable tumours, tumour size was measured every 3 or 4 days using calipers, and tumour volume was calculated according to the formula length × width^2^ × 0.5236. Mice were euthanized in a 7.5%-CO_2_ chamber when they met the institutional euthanasia criteria for tumour size and overall health. The tumours were harvested, photographed, weighed and subjected to other analyses.

### Statistical analysis

Statistical analyses were performed using SPSS version 18.0 (SPSS Inc., Chicago, IL). Unless stated otherwise, all data are presented as the mean±s.d. IHC scores were compared by one-way ANOVA and an independent *t*-test. Spearman's rank correlation analysis was used to evaluate the associations between pAKT, pmTOR, PTEN and MKRN1 expression. Survival curves were estimated by the Kaplan–Meier analysis, and survival curves between groups were compared using the log-rank test. Multivariate analysis was performed using the Cox proportional hazards model to identify independent predictors of survival after adjustment by relevant clinical covariates. A value of *P*<0.05 was considered statistically significant. The significance of differences between groups was also validated by one-way ANOVA and the unpaired two-tailed *t*-test using Prism (version 7.0; GraphPad).

## Additional information

**How to cite this article:** Lee, M.-S. *et al*. PI3K/AKT activation induces PTEN ubiquitination and destabilization accelerating tumourigenesis. *Nat. Commun*. 6:7769 doi: 10.1038/ncomms8769 (2015).

## Supplementary Material

Supplementary Information Supplementary Figures 1-14 and Supplementary Tables 1-2
