# Author Correction: PI3K/AKT activation induces PTEN ubiquitination and destabilization accelerating tumourigenesis

**DOI:** 10.1038/s41467-020-20178-0

**Published:** 2020-12-01

**Authors:** Min-Sik Lee, Man-Hyung Jeong, Hyun-Woo Lee, Hyun-Ji Han, Aram Ko, Stephen M. Hewitt, Jae-Hoon Kim, Kyung-Hee Chun, Joon-Yong Chung, Cheolju Lee, Hanbyoul Cho, Jaewhan Song

**Affiliations:** 1 Department of Biochemistry, College of Life Science and Biotechnology, Yonsei University, Seoul, 120-749 Republic of Korea; 2 Department of Biochemistry and Molecular Biology, Yonsei University College of Medicine, Seoul, 120-752 Republic of Korea; 3 Experimental Pathology Laboratory, Center for Cancer Research, National Cancer Institute, NIH MSC 1500, Bethesda, MD 20892 USA; 4 Department of Obstetrics and Gynecology, Gangnam Severance Hospital, Yonsei University College of Medicine, Seoul, 135-720 Republic of Korea; 5 Institute of Women’s Life Medical Science, Yonsei University College of Medicine, Seoul, 120-752 Republic of Korea; 6 BRI, Korea Institute of Science and Technology, Seoul, 136-791 Korea

Correction to: *Nature Communications* 10.1038/ncomms8769, published online 17 July 2015.

In this Article, there is an error in Fig. 7, in which the PTEN western blot in the IP fraction of Fig. 7c is inadvertently reproduced in the IP fraction of Fig. 7b. This error does not affect the scientific validity of the conclusions, and has not been corrected in the original version of the Article. The correct Fig. 7 is present below.

**Figure 1:**
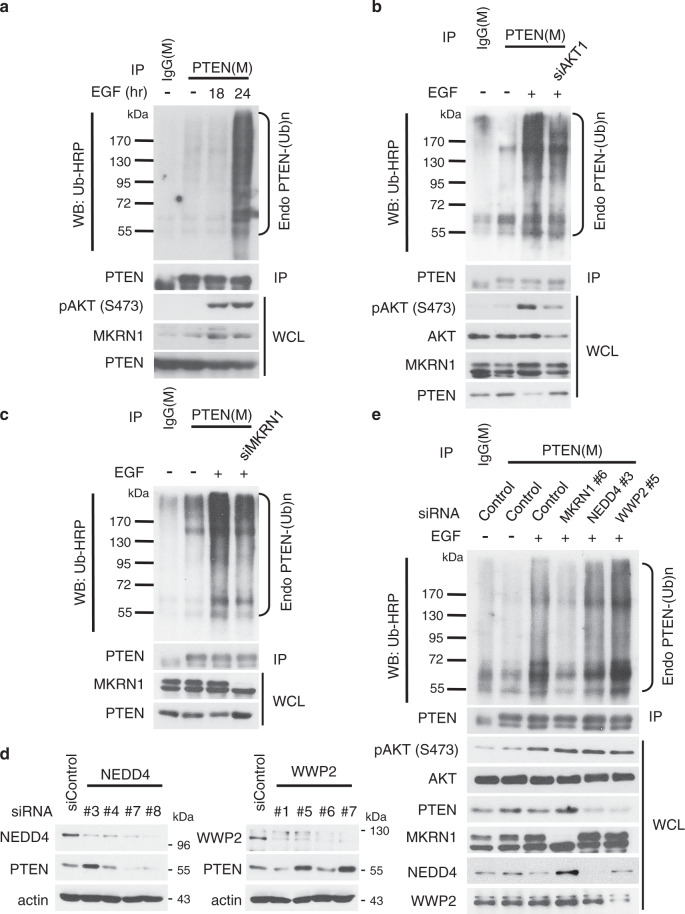
Fig. 7

