# Supplementary material for: PI3K/AKT activation induces PTEN ubiquitination and destabilization accelerating tumourigenesis
Source: Nat Commun. 2015 Jul 17;6:7769. doi: 10.1038/ncomms8769 (PMC4518267; doi:10.1038/ncomms8769)
Supplement: Supplementary Information — Supplementary Figures 1-14 and Supplementary Tables 1-2 [file ncomms8769-s1.pdf]

Supplementary Figures

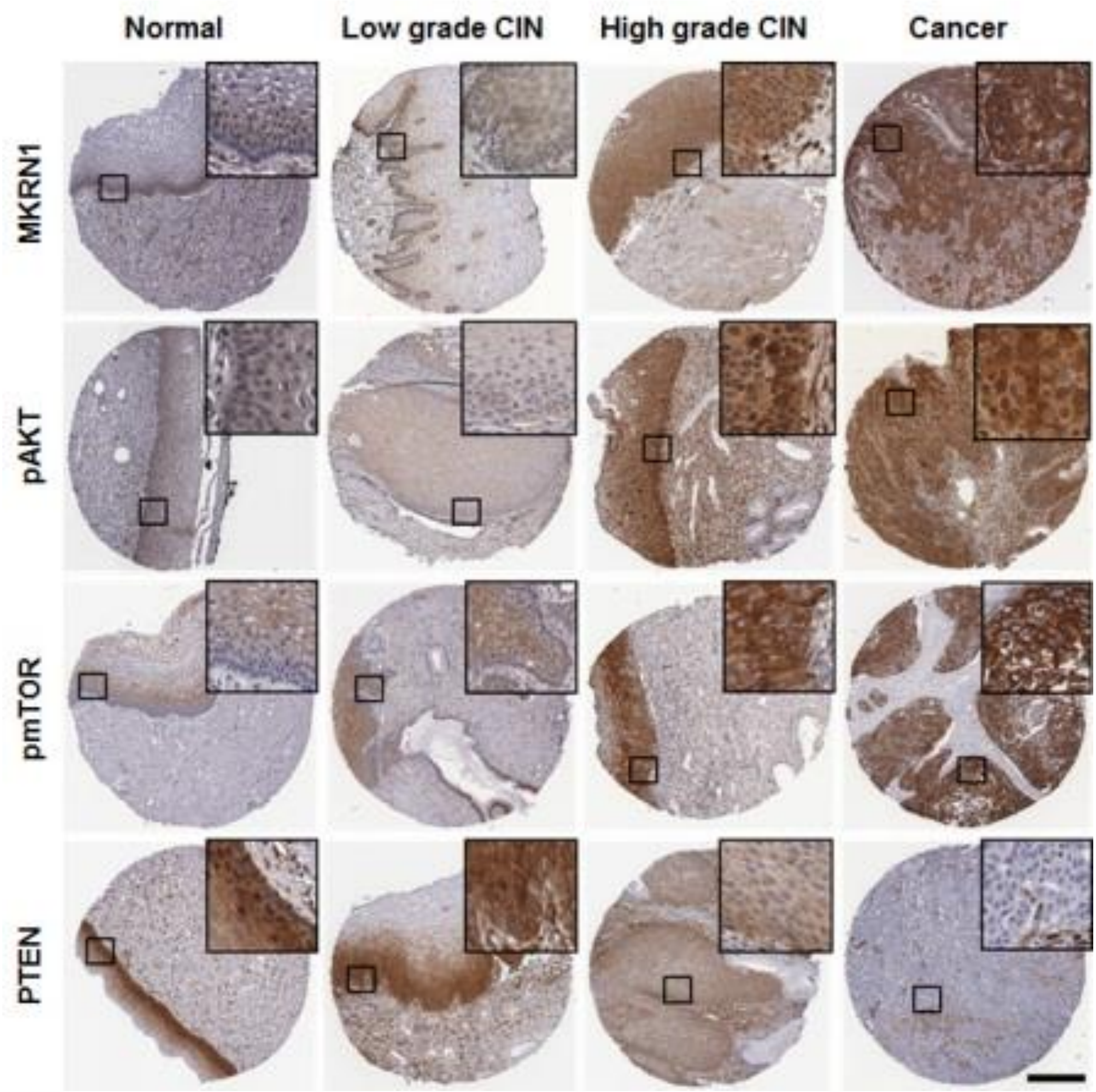

Supplementary Figure 1. Representative images of immunohistochemical staining of MKRN1, pAKT, pmTOR, and PTEN in human cervical cancer cells. The boxed regions are displayed at high magnification in the inset. Bars: 200 μm.

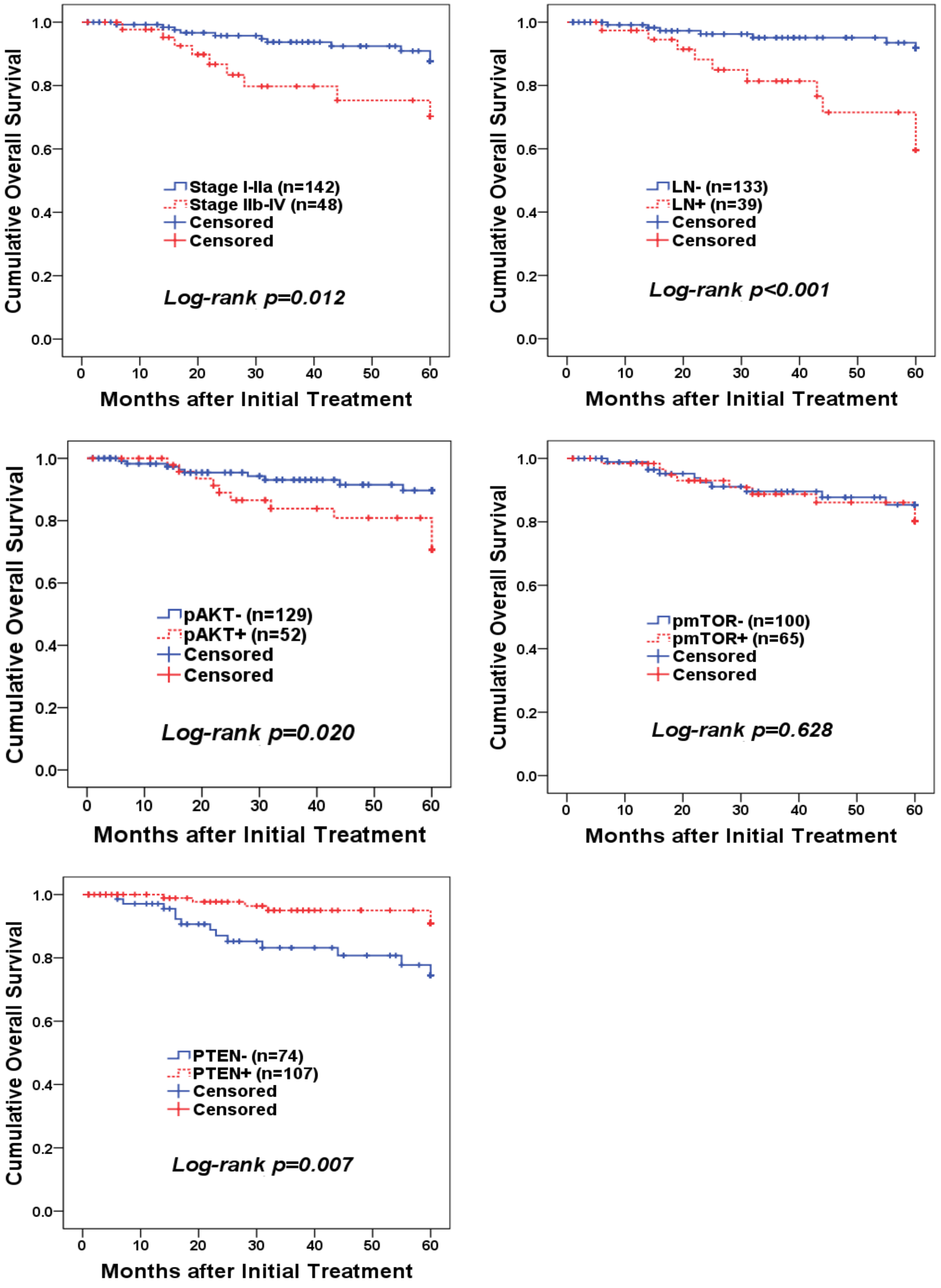

Supplementary Figure 2. Survival analysis of pAKT, pmTOR, and PTEN. The Kaplan–Meier plots show the overall survival for cervical cancer patients categorized by tumor stage, LN metastasis, and expression of pAKT, pmTOR, or PTEN. The high pAKT expression (IHC histoscore ≥ 8) group had a shorter overall survival (mean = 53.3 versus 56.7 months, p = 0.020), whereas the low PTEN expression (IHC histoscore < 1) group had the shortest overall survival (mean = 52.4 versus 58.1 months, p = 0.007). Data were analyzed using a one-way ANOVA and independent t-test.

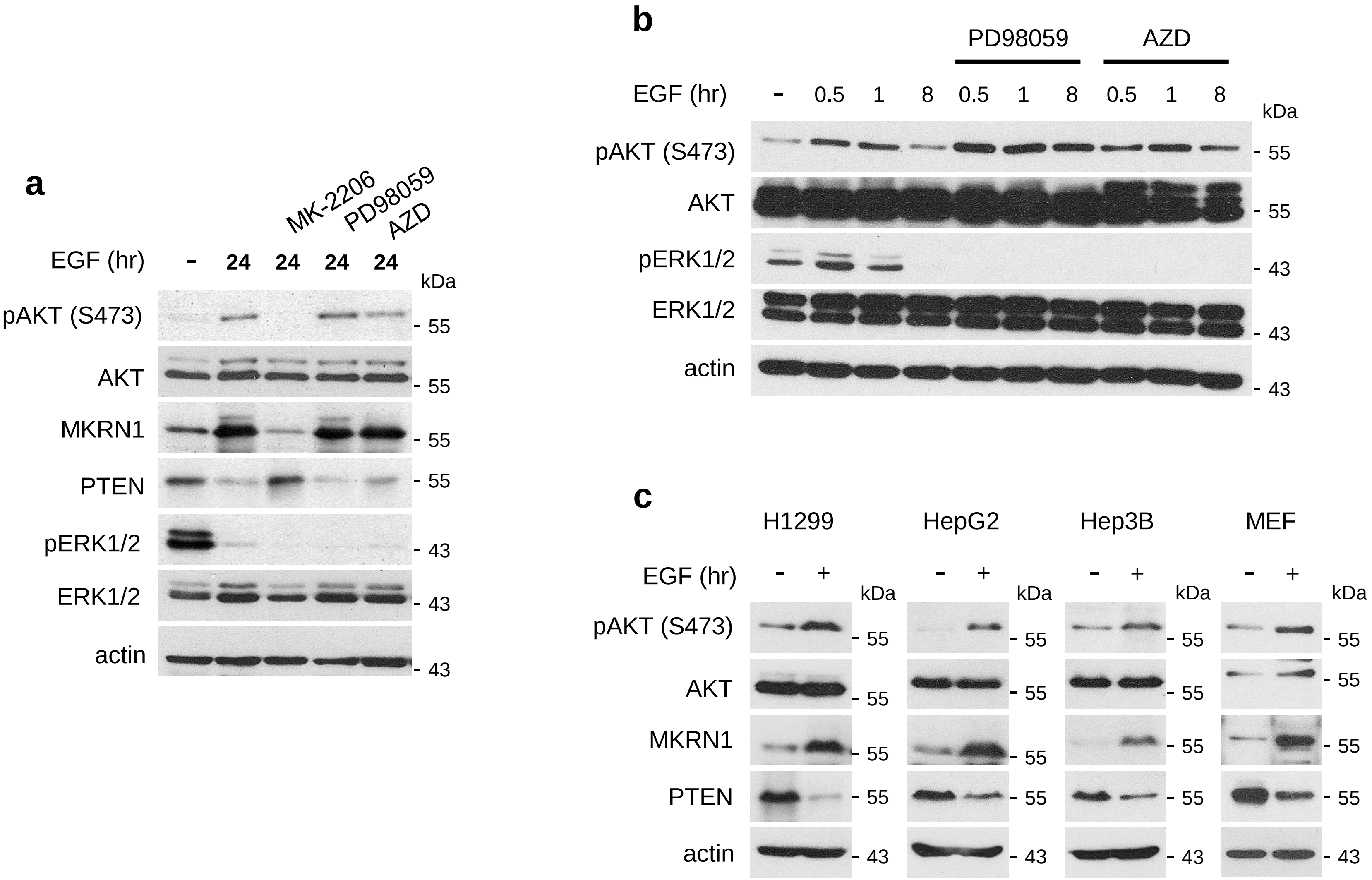

Supplementary Figure 3. EGF-driven regulation of MKRN1 and PTEN. (**a,b**) The MEK inhibitors do not have much effect on the EGF-driven regulation of MKRN1 and PTEN. After serum starvation, ME-180 cells were treated with EGF (100 ng/ml) and MK-2206 (1 μM, AKT inhibitor) or PD98059 (20 μM, the MEK inhibitor) or AZD6244 (1 μM, the MEK inhibitor) for the indicated time. (**c**) The effect of EGF treatment on the up- and down-regulation of MKRN1 and PTEN, respectively, in H1299, HepG2, and Hep3B cells and MEFs.

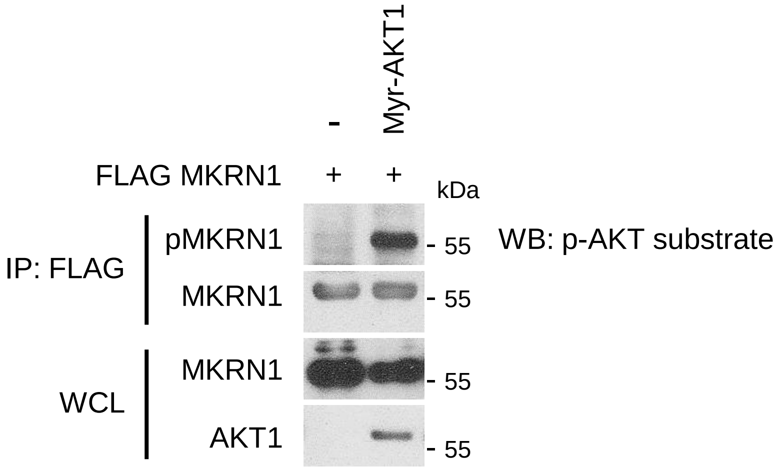

Supplementary Figure 4. The use of a phospho-AKT substrate antibody indicates the MKRN1 phosphorylation. H1299 cells were co-transfected with FLAG-MKRN1 and HA-tagged Myr-AKT. Ectopically expressed MKRN1 was immunoprecipitated using an anti-FLAG antibody, and phospho-MKRN1 was detected using a phospho-AKT substrate (p-RXRXXS/T) antibody (IP panel); WCL was immunoblotted.

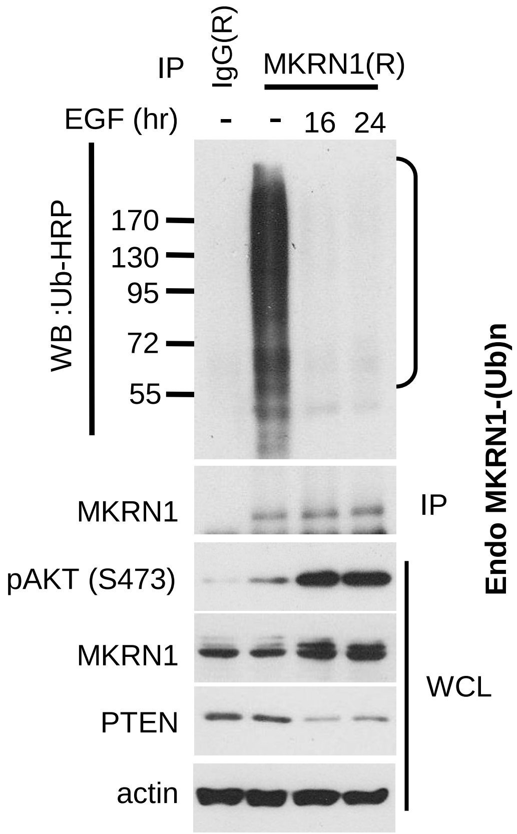

Supplementary Figure 5. Endogenous MKRN1 ubiquitination upon EGF treatment. ME-180 cells were stimulated with EGF and then were treated with MG132 (10 μM) for 4 h. Lysates were immunoprecipitated with an anti-MKRN1 antibody, followed by immunoblotting with an HRP-conjugated anti-Ub antibody under denaturing conditions.

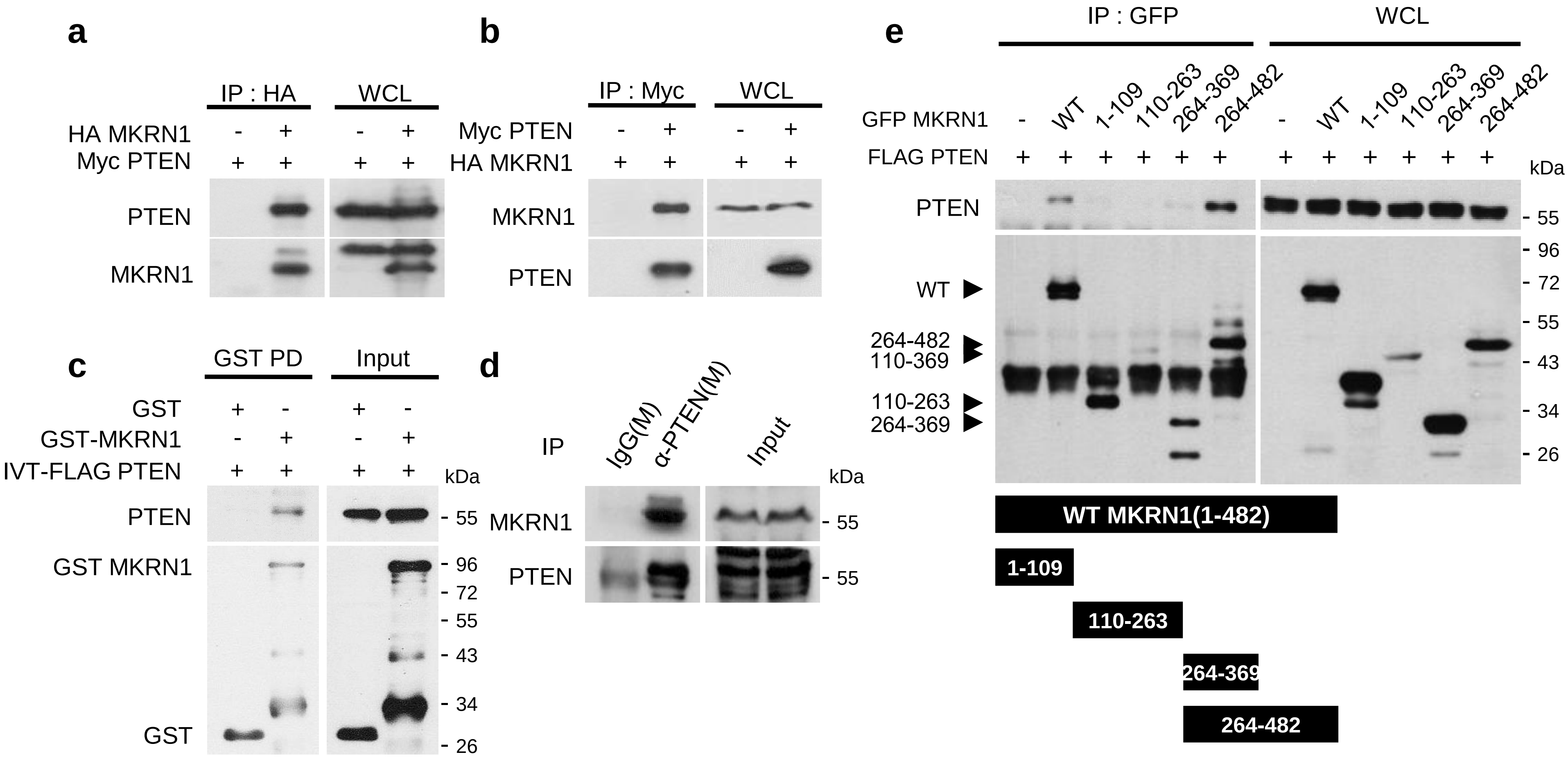

Supplementary Figure 6. Direct interaction of MKRN1 and PTEN. (**a,b**) MKRN1 binds to PTEN. HEK293T cells were transfected with the indicated plasmids and immunoprecipitated with either anti-HA or anti-Myc antibodies followed by immunoblotting. (**c**) The interaction of MKRN1 and PTEN in vitro. After incubation of bacterially expressed GST-MKRN1 and in vitro-translated FLAG-PTEN proteins, GST-MKRN1 was pulled down by glutathione Sepharose beads. (**d**) The interaction of endogenous MKRN1 and PTEN. HeLa cell lysates were immunoprecipitated with an anti-PTEN antibody, followed by immunoblotting. (**e**) The C-terminus of MKRN1 is required for its interaction with PTEN. Ectopically expressed truncated GFP-MKRN1 constructs and FLAG-PTEN were immunoprecipitated with an anti-GFP antibody. Schematic models represent the truncated MKRN1 constructs.

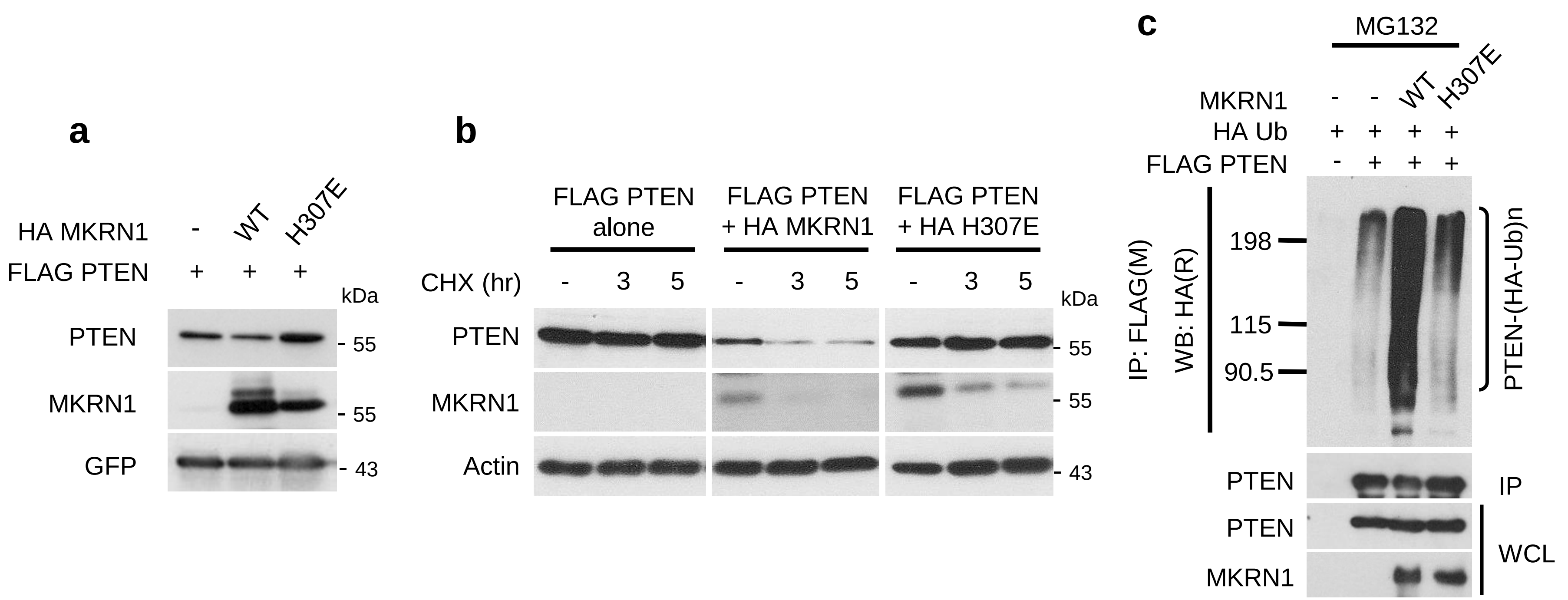

Supplementary Figure 7. The E3 ligase activity of MKRN1 is required for its regulation of PTEN. (**a**) Overexpression of HA-MKRN1 WT, but not H307E, results in the degradation of ectopically expressed FLAG-PTEN in H1299 cells. (**b**) H1299 cells were transfected with FLAG-PTEN in the absence or presence of HA-MKRN1 WT or H307E and then were treated with CHX at the indicated time points. (**c**) MKRN1 WT, but not H307E, induces PTEN ubiquitination. H1299 cells were transfected with the indicated plasmids, followed by treatment with MG132 (10 μM) for 6 h. HA-tagged ubiquitinated PTEN was purified by immunoprecipitation using an anti-FLAG antibody in 1% SDS buffer, followed by immunoblotting using an anti-HA antibody.

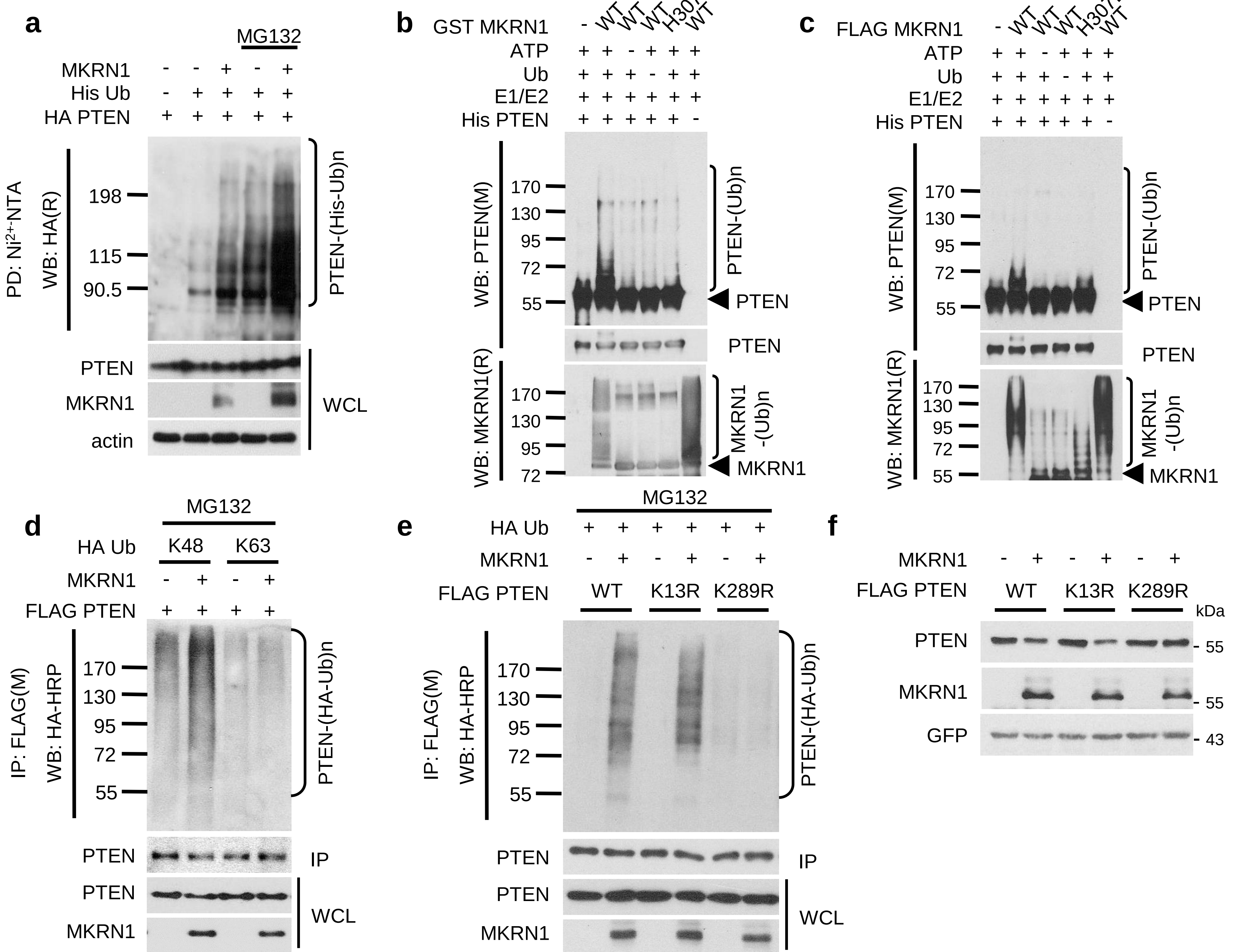

a
MG132
MKRN1 - - + - +
His Ub - + + + +
HA PTEN + + + + +
PD: Ni2+-NTA
WB: HA(R)
198
115
90.5
PTEN-(His-Ub)n
PTEN
MKRN1
actin
WCL
b
GST MKRN1 - WT WT WT H307E WT
ATP + + - + + +
Ub + + + - + +
E1/E2 + + + + + +
His PTEN + + + + + -
WB: PTEN(M)
170
130
95
72
55
PTEN-(Ub)n
PTEN
PTEN
WB: MKRN1(R)
170
130
95
72
MKRN1 -(Ub)n
MKRN1
c
FLAG MKRN1 - WT WT WT H307E WT
ATP + + - + + +
Ub + + + - + +
E1/E2 + + + + + +
His PTEN + + + + + -
WB: PTEN(M)
170
130
95
72
55
PTEN-(Ub)n
PTEN
PTEN
WB: MKRN1(R)
170
130
95
72
55
MKRN1 -(Ub)n
MKRN1
d
MG132
HA Ub K48 K63
MKRN1 - + - +
FLAG PTEN + + + +
IP: FLAG(M)
WB: HA-HRP
170
130
95
72
55
PTEN-(HA-Ub)n
PTEN
IP
PTEN
MKRN1
WCL
e
MG132
HA Ub + + + + + +
MKRN1 - + - + - +
FLAG PTEN WT K13R K289R
IP: FLAG(M)
WB: HA-HRP
170
130
95
72
55
PTEN-(HA-Ub)n
PTEN
IP
PTEN
MKRN1
WCL
f
MKRN1 - + - + - +
FLAG PTEN WT K13R K289R
kDa
PTEN - 55
MKRN1 - 55
GFP - 43

Supplementary Figure 8. MKRN1 ubiquitinates PTEN at the lysine 289 site via K48 linkage. (**a**) MKRN1 ubiquitinates PTEN. To identify PTEN ubiquitination, H1299 cells were transfected with His-Ub and the indicated plasmids, followed by treatment with MG132 (10 μM) for 6 h. His-tagged ubiquitinated PTEN was purified using Ni2+-NTA beads. (**b,c**) Bacterially expressed His-PTEN was incubated with E1, E2, and ubiquitin (Ub) in the absence or presence of ATP along with GST, GST-MKRN1, or H307E (bacterially purified) or FLAG-MKRN1 or H307E proteins (purified from HEK293T cells), respectively, as indicated for the in vitro ubiquitination of PTEN. (**d**) MKRN1 promotes K48-linked polyubiquitination of PTEN. H1299 cells were co-transfected with FLAG-PTEN, MKRN1 and HA-Ub K48-only mutant (K48) or K63-only mutant (K63). HA-tagged ubiquitinated PTEN was identified by immunoprecipitation using an anti-FLAG antibody in 1% SDS buffer, followed by immunoblotting using an anti-HA antibody. (**e**) MKRN1 targets the K289 site on PTEN for ubiquitination. H1299 cells were co-transfected with HA-Ub, FLAG-PTEN (WT, K13R or K289R) and MKRN1 as indicated. HA-tagged ubiquitinated PTEN was identified as described in **d**. (**f**) PTEN K289R mutant was protected from MKRN1-mediated degradation.

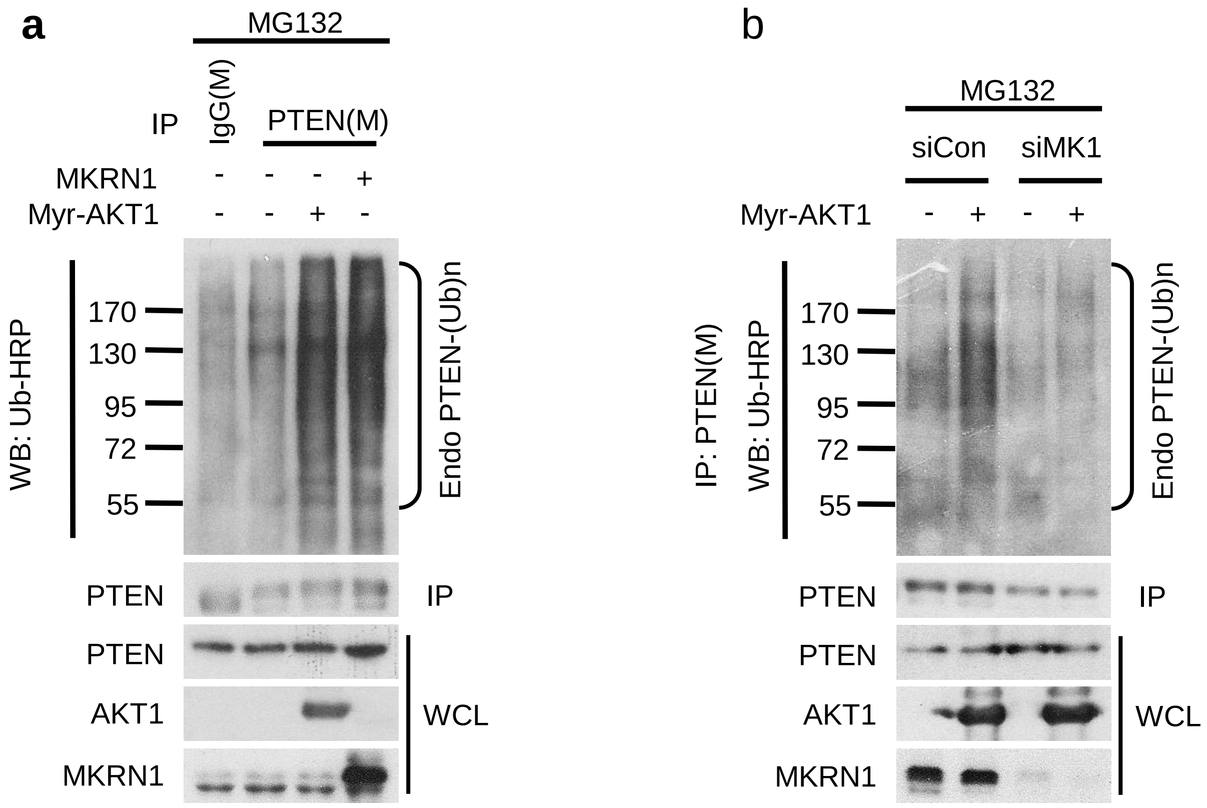

Supplementary Figure 9. Myr-AKT induces MKRN1-mediated PTEN ubiquitination. (**a,b**) ME-180 cells (**a**) or siControl- or siMKRN1-transduced ME-180 cells (**b**) were transfected with Myr-AKT or MKRN1 as indicated and treated with MG132 (10 μM). Lysates were immunoprecipitated with an anti-PTEN antibody, and ubiquitinated PTEN was then immunoblotted using an HRP-conjugated anti-Ub antibody under denaturing conditions.

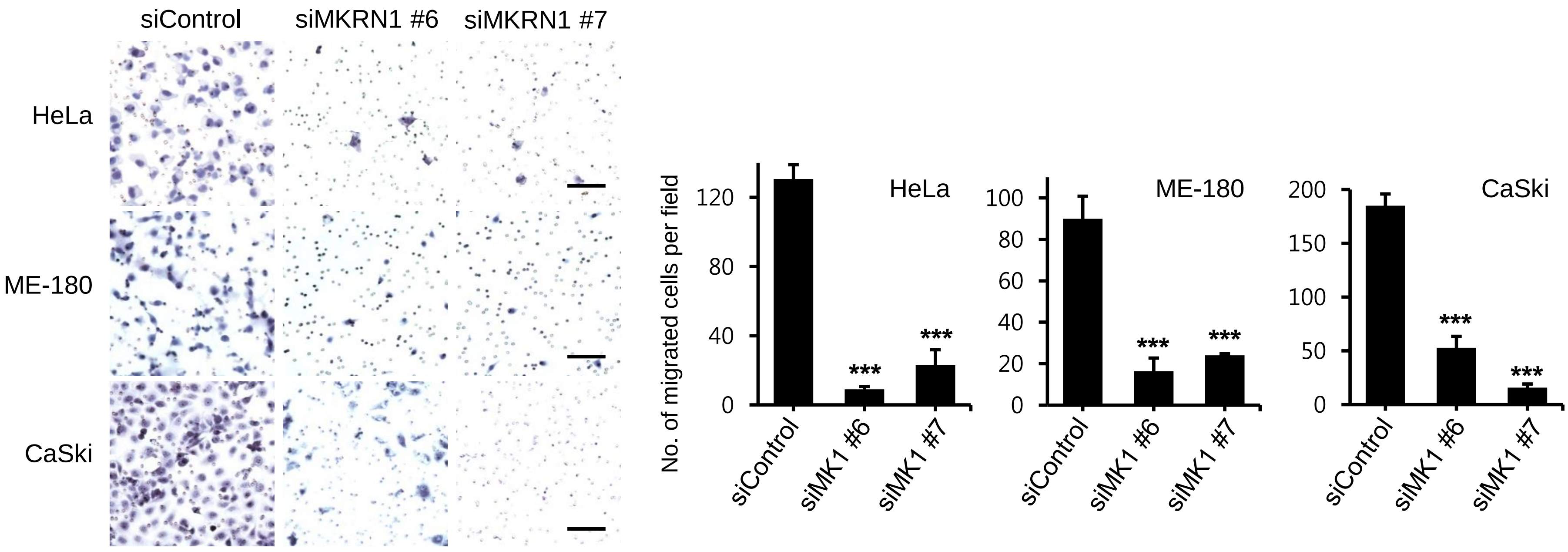

Supplementary Figure 10. MKRN1 ablation reduces cervical cancer cell mobility. Using a collagen-coated Transwell chamber, cervical cancer cell lines transfected with two types of MKRN1 siRNA (siMKRN1 #6, siMKRN1 #7) were analyzed using a migration assay. The photographic images represent H&E-stained migratory cells (left panel, magnification: ×200, scale bar: 50 μm). The average number of migratory cells per field of view was quantified and described (right panel, data shown are means ± s.d.; n = 3) ***$p < 0.001$ (Student's t test).

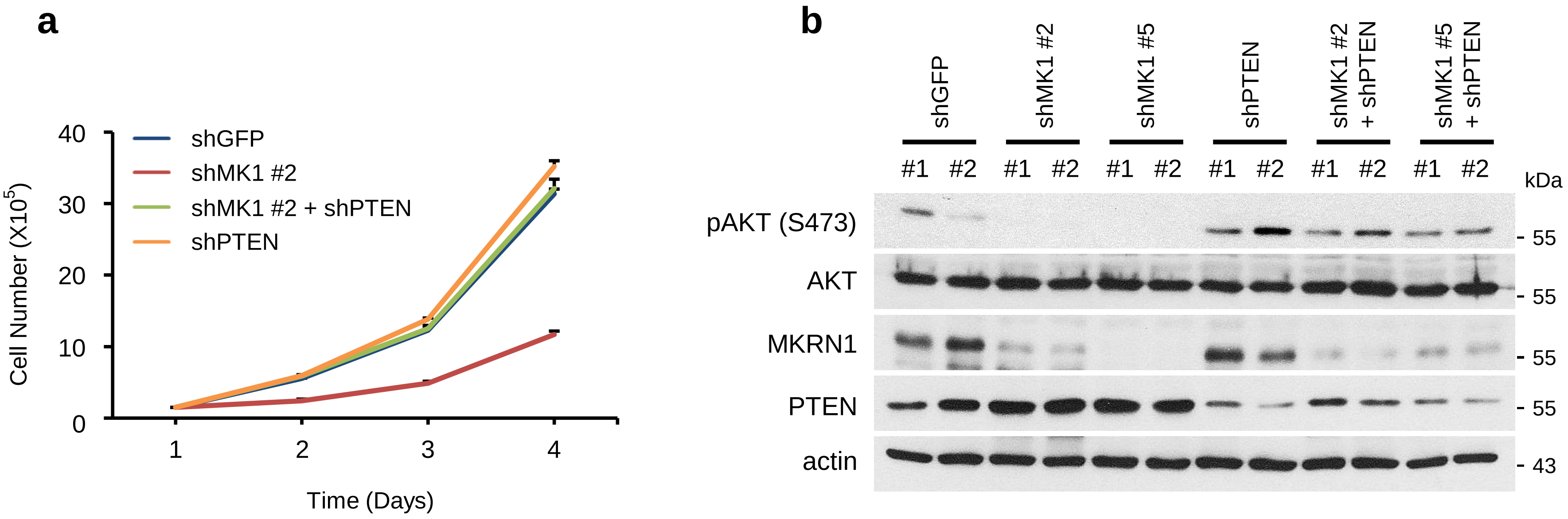

Supplementary Figure 11. Growth curve of ME-180 cells stably expressing MKRN1 shRNA, PTEN shRNA or both, and the molecular profiles of xenografted tumor samples. (**a**) Ablation of PTEN rescued the cell growth retardation induced by MKRN1 depletion. The same amount of ME-180 stably expressing shGFP or MKRN1 shRNA or PTEN shRNA was plated and counted 1, 2, 3 and 4 days later. Error bars indicate s.d. n = 3. (**b**) Immunoblotting of p-AKT, AKT, MKRN1, and PTEN in tumor lysates from the xenografts shown in Figure 8c at day 38 after implantation.

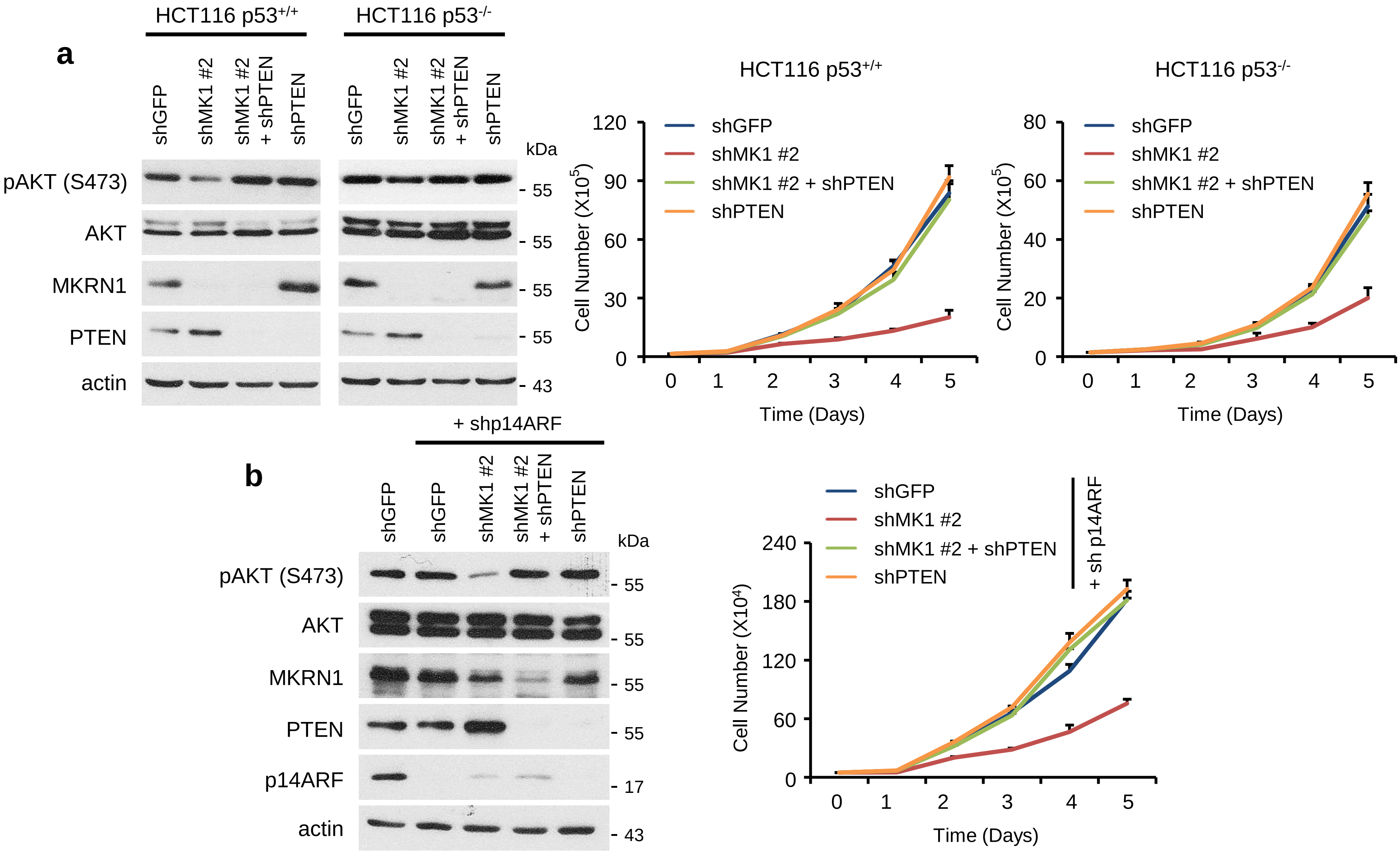

Supplementary Figure 12. Depletion of PTEN completely reversed the tumor-suppressive effects achieved by MKRN1 knockdown in p53- and p14ARF-depleted cancer cells. (**a**) Immunoblotting of p-AKT, AKT, MKRN1, and PTEN in cell lysates from HCT116 p53$^{+/+}$ or p53$^{-/-}$ cells stably expressing MKRN1 shRNA or PTEN shRNA (left panel). *In vitro* cell growth (right panel, Error bars indicate s.d. n = 3) (**b**) The molecular profiles of ME-180 cells stably expressing shRNAs as indicated (left panel). *In vitro* cell growth (right panel, Error bars indicate s.d. n = 3).

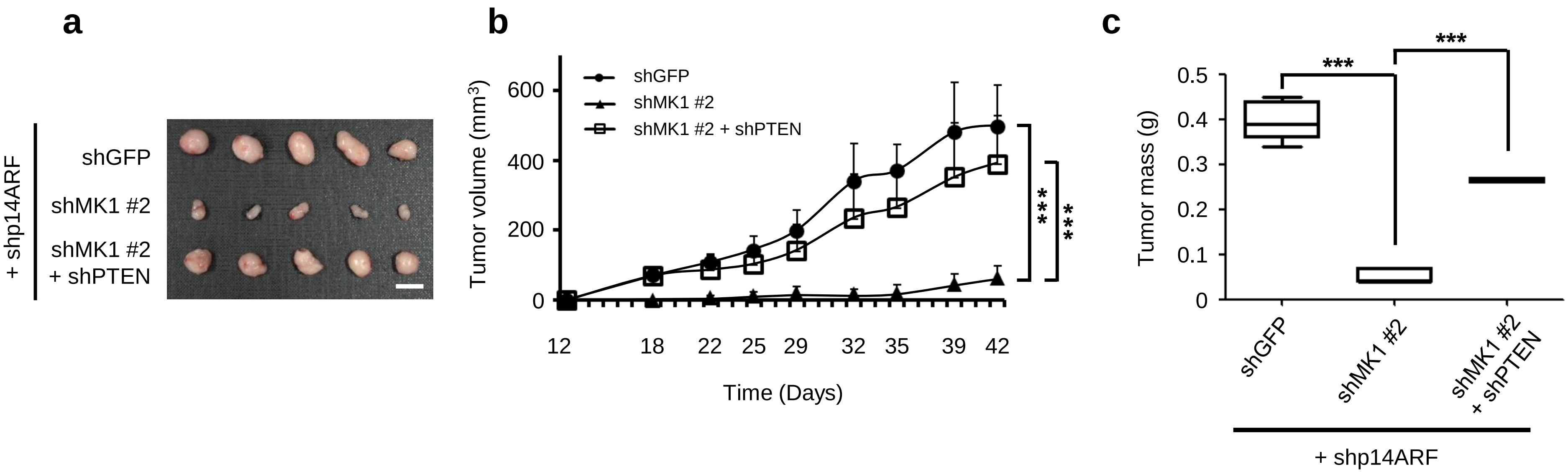

Supplementary Figure 13. The tumor-suppressive effects of MKRN1 depletion is reversed upon PTEN depletion in the p14ARF-abrogated cervical cancer cell line ME-180. (**a**) Images depict tumors at day 42 after the subcutaneous injection of 2 × 10^6 ME-180 cells stably expressing shARF and shGFP or MKRN1 shRNA (shMK1 #2) or shMK1 #2 and PTEN shRNA (shPTEN). Bars, 1 cm. (**b**) The graph shows tumor growth. (**c**) A tumor mass is shown at day 42 after implantation. The data shown in (**b**) and (**e**) are the means ± s.d. n = 5 mice per group in (**a**-**c**). *p < 0.05, **p < 0.01, and ***p < 0.001, based on Student’s t test.

Fig. 1b

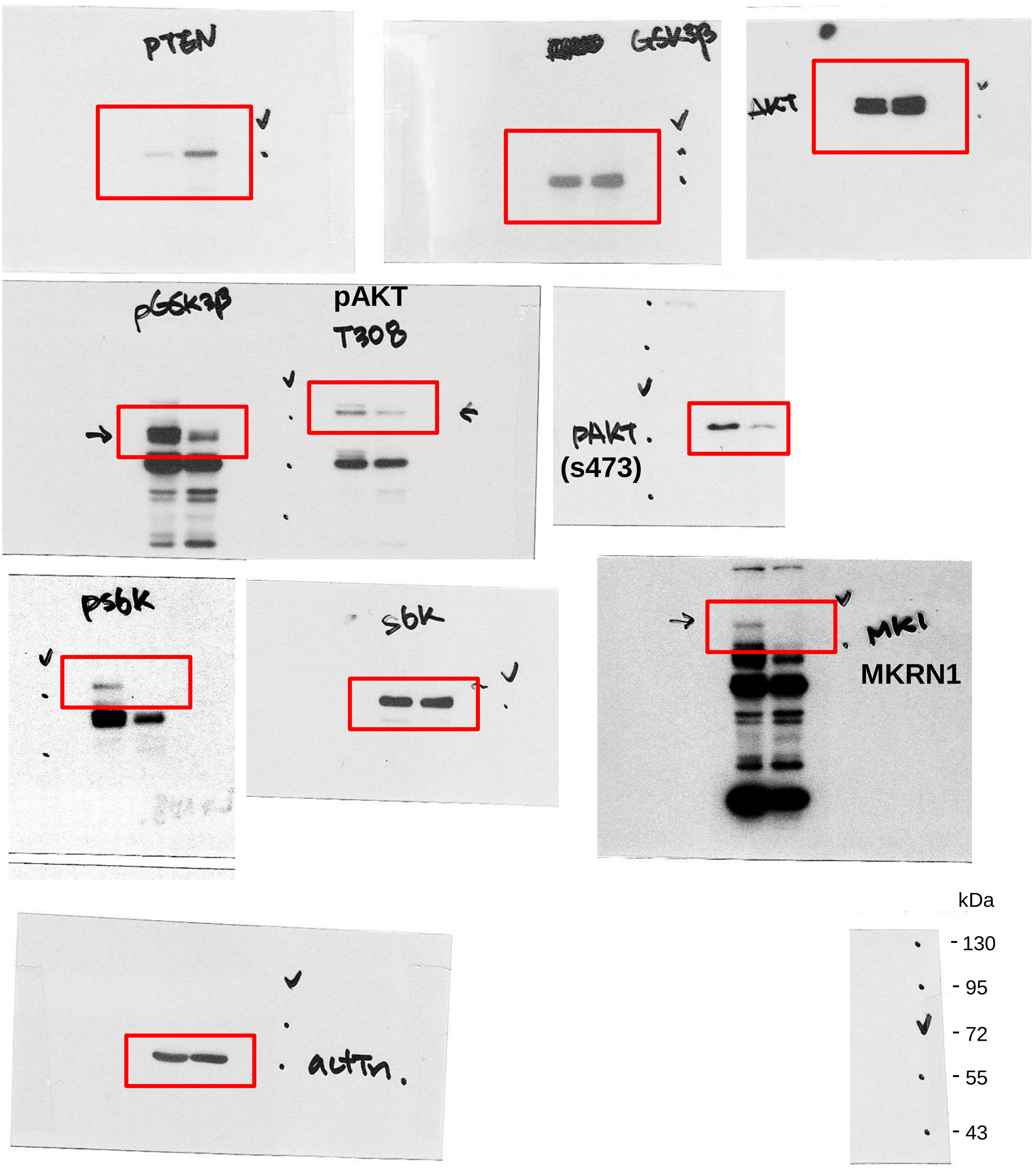

Supplementary Figure 14. Uncropped blots

(a) Blots from Figure 1.

Fig. 1c

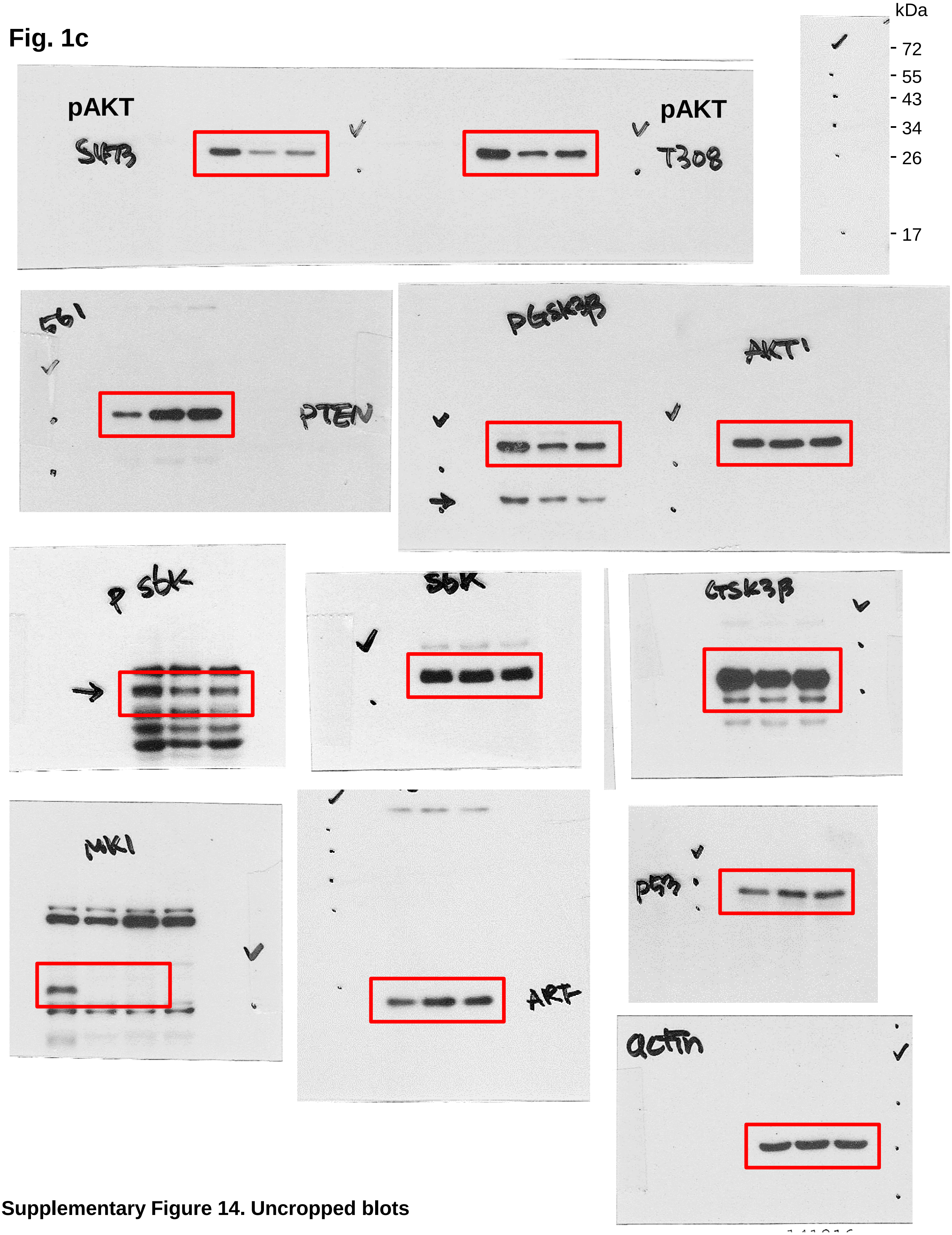

Supplementary Figure 14. Uncropped blots

(a) Blots from Figure 1.

Fig. 1f

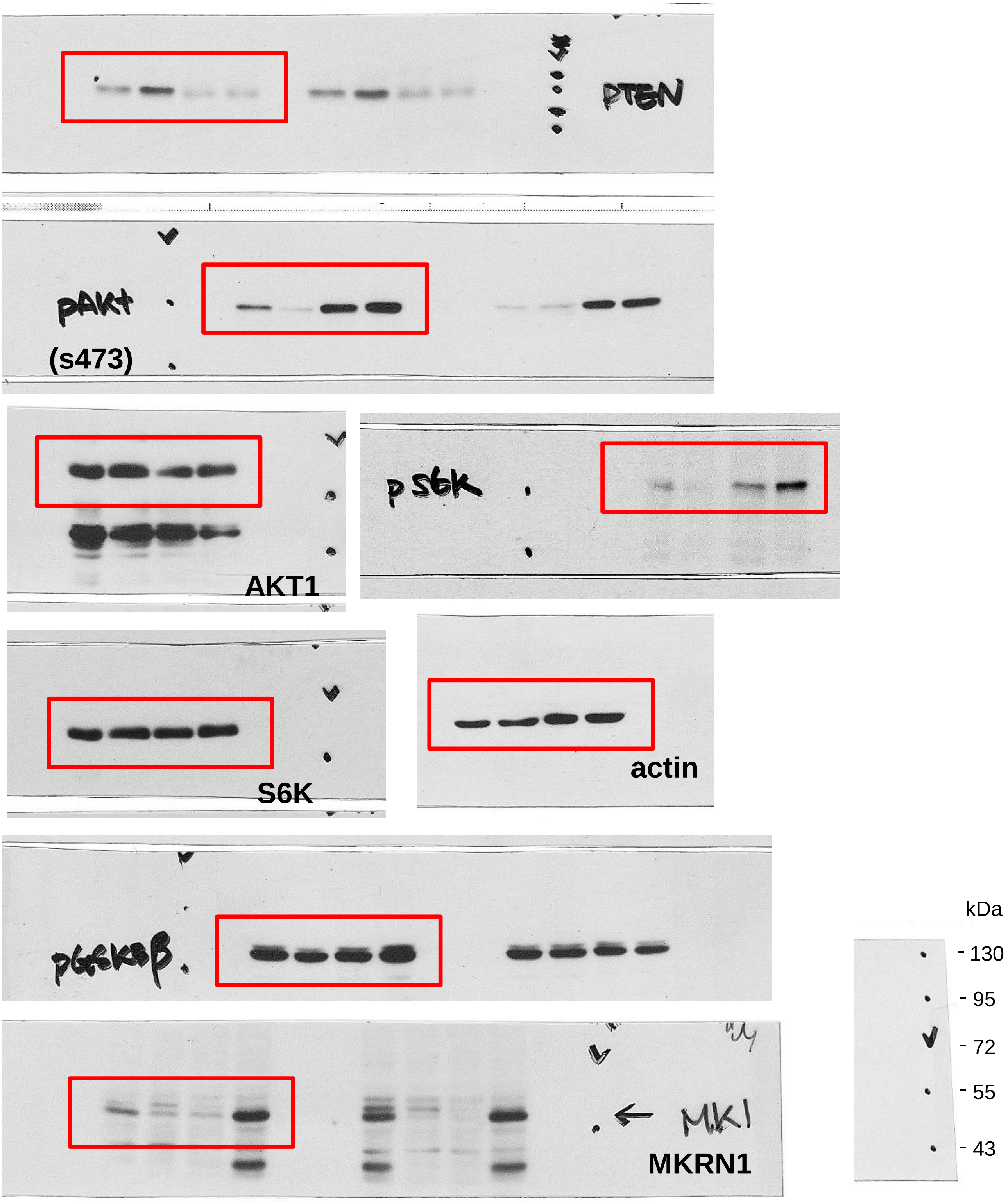

Supplementary Figure 14. Uncropped blots

(a) Blots from Figure 1.

Fig. 3a

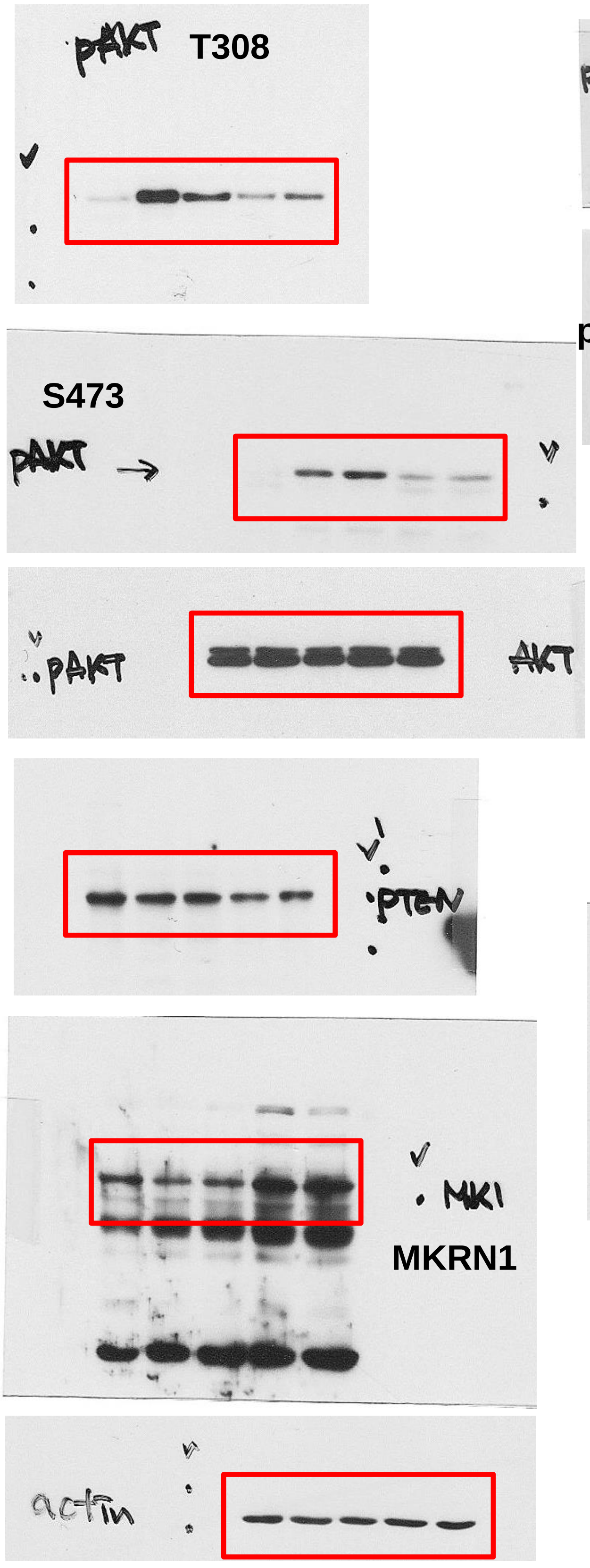

Fig. 3d

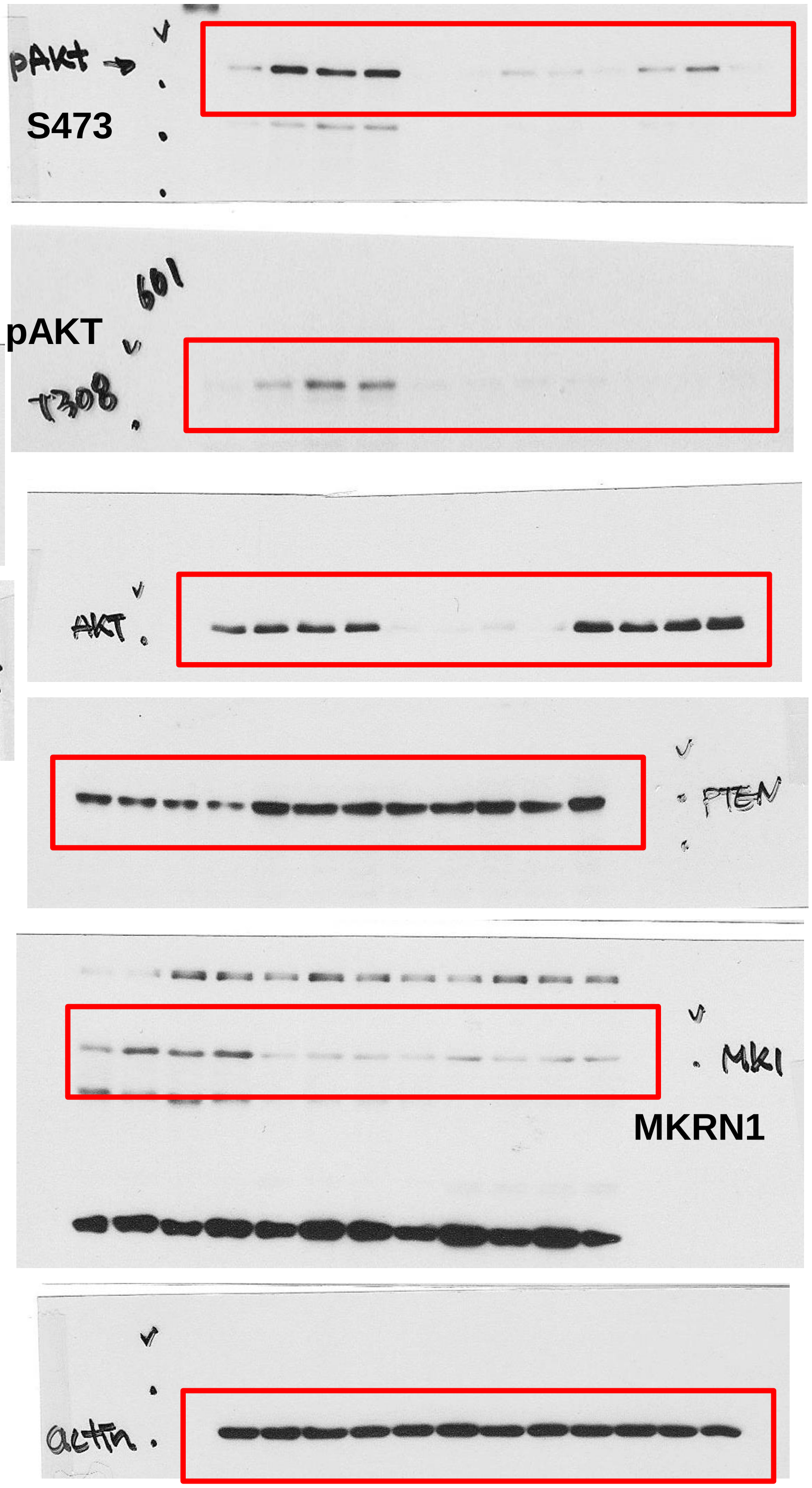

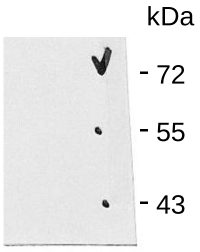

Supplementary Figure 14. Uncropped blots

(b) Blots from Figure 3.

Fig. 3e

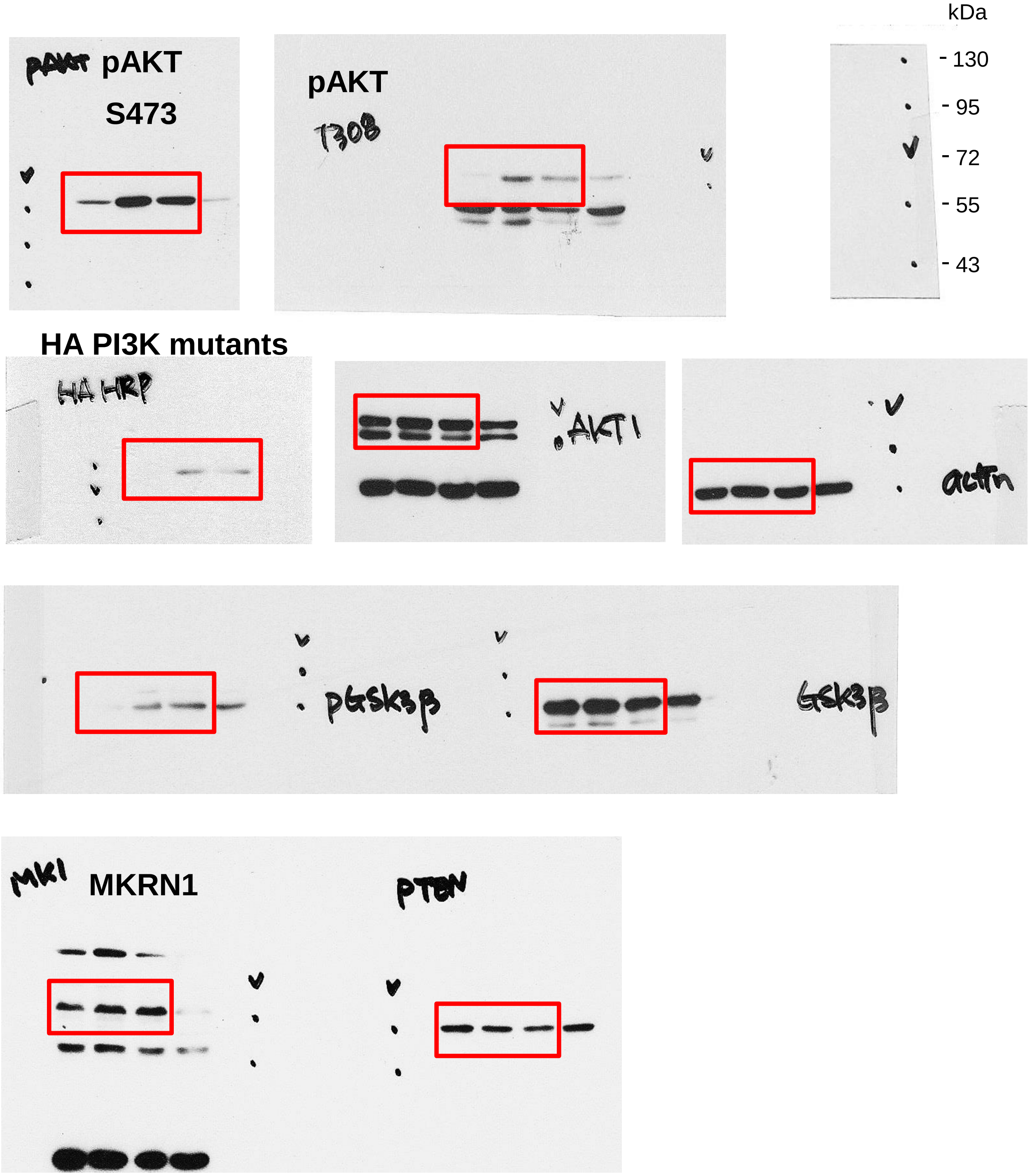

Supplementary Figure 14. Uncropped blots

(b) Blots from Figure 3.

Fig. 4c

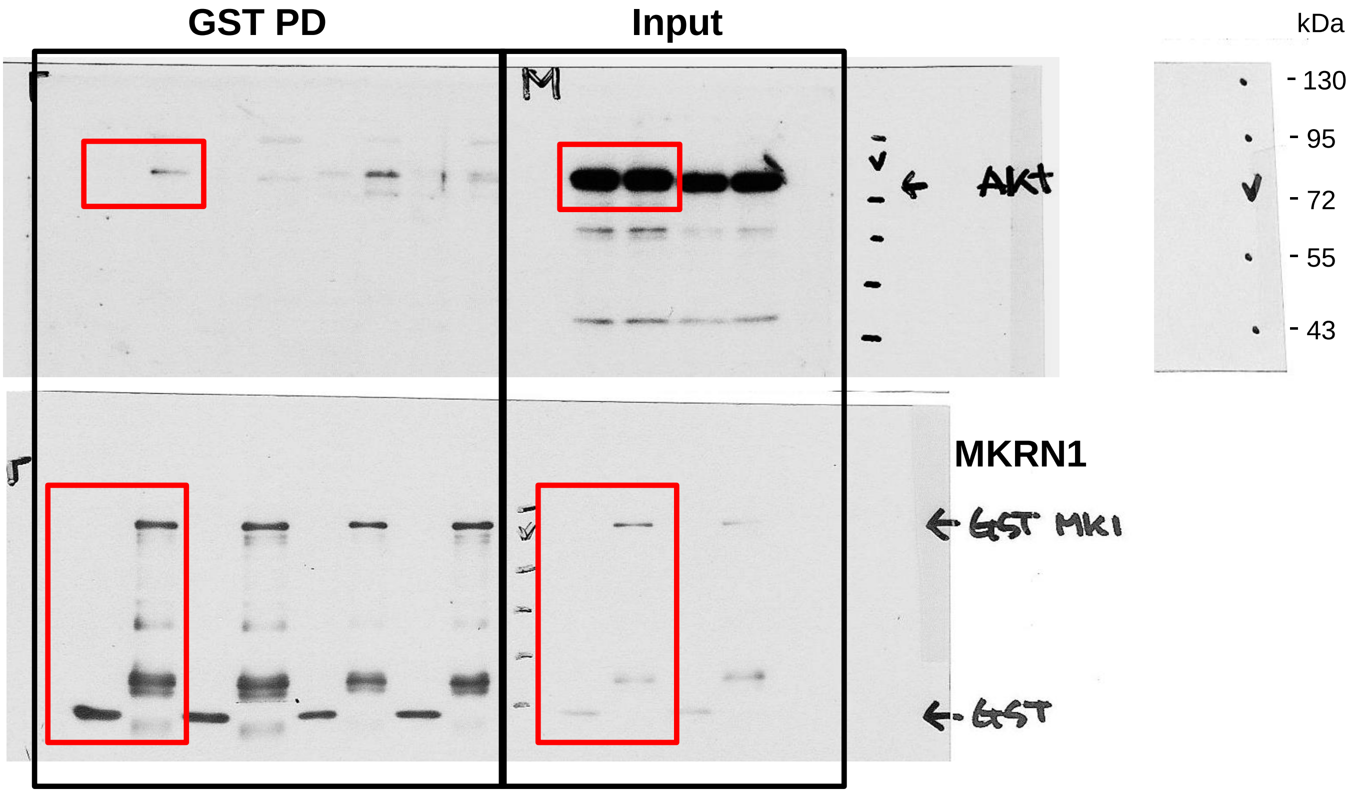

Fig. 4d

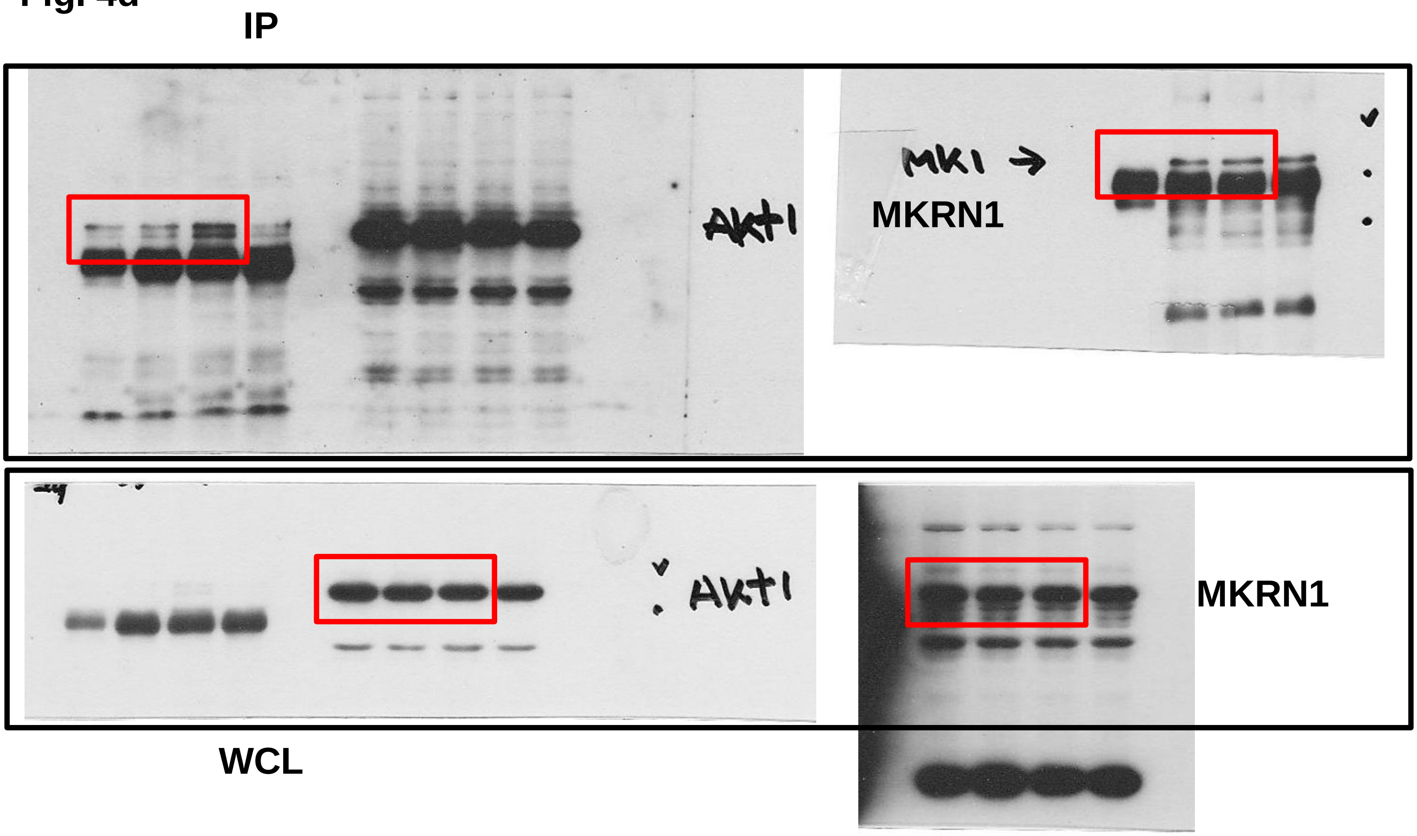

Supplementary Figure 14. Uncropped blots

(c) Blots from Figure 4.

Fig. 4g

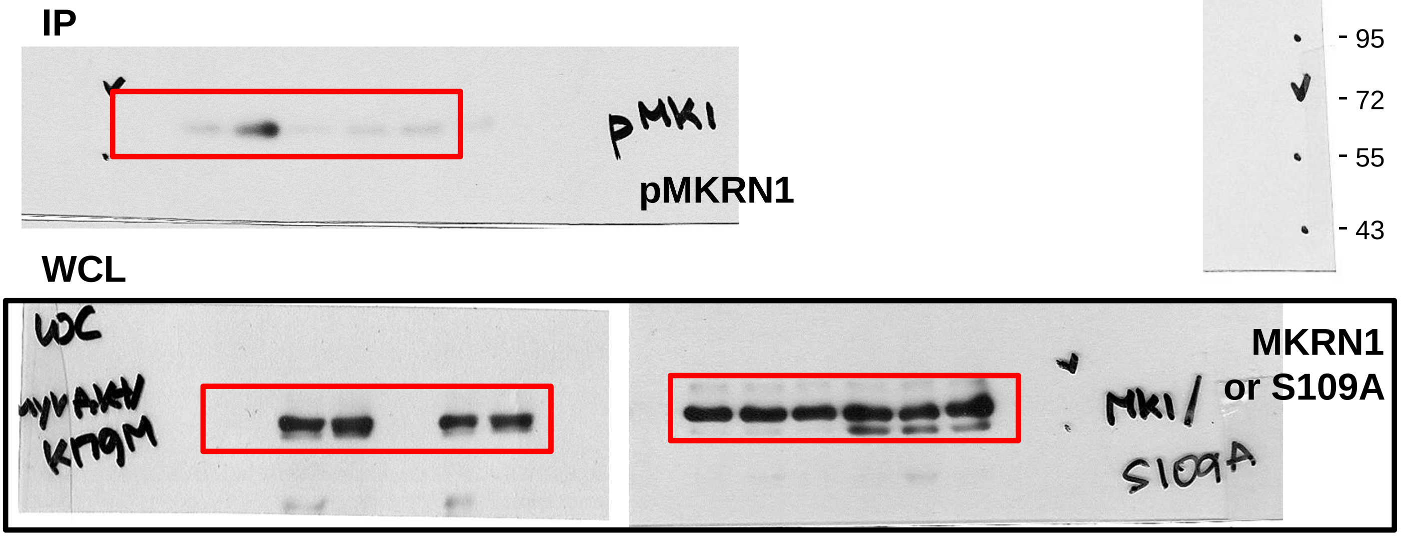

Fig. 4h

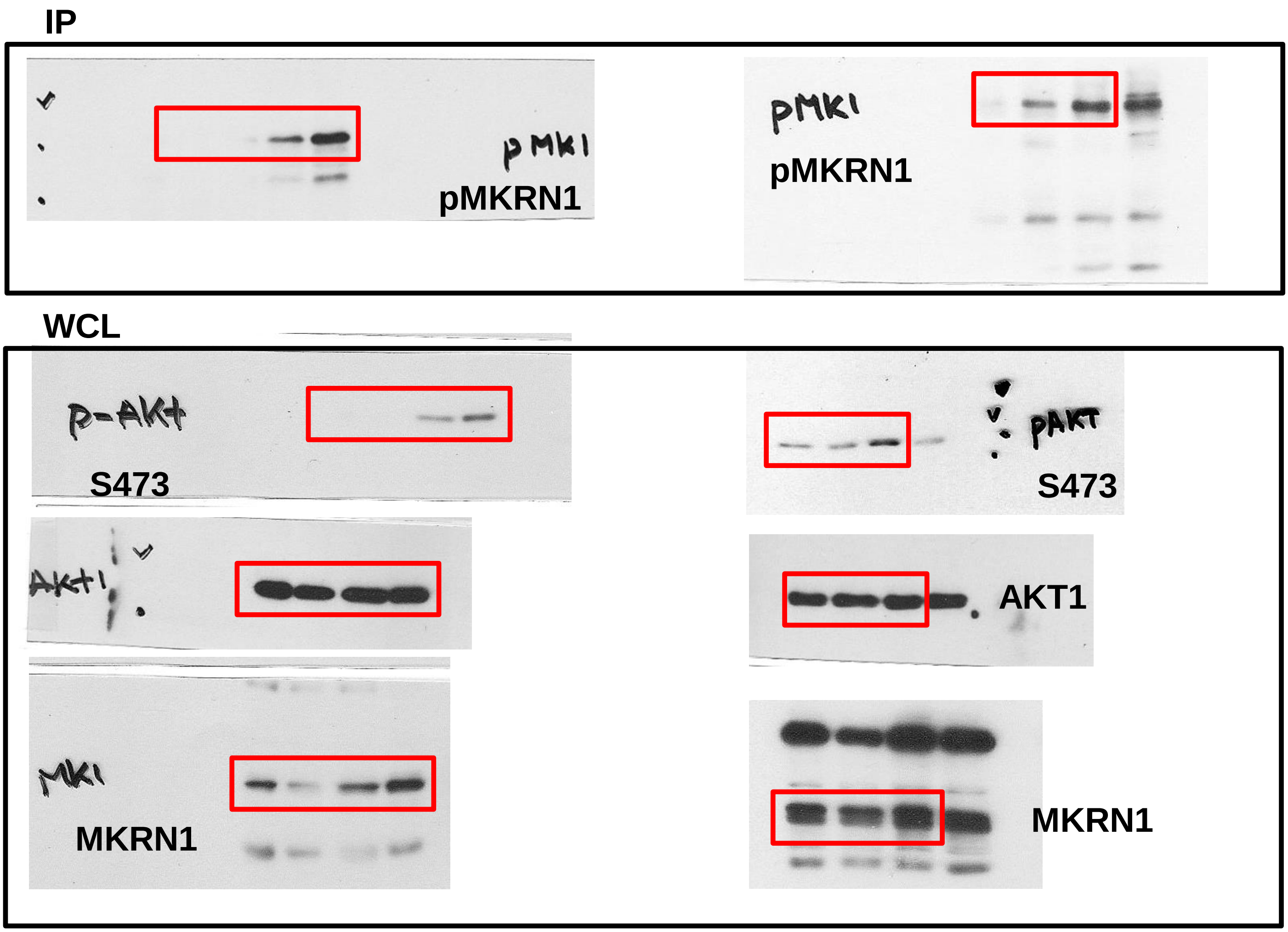

Supplementary Figure 14. Uncropped blots

(c) Blots from Figure 4.

Fig. 5a

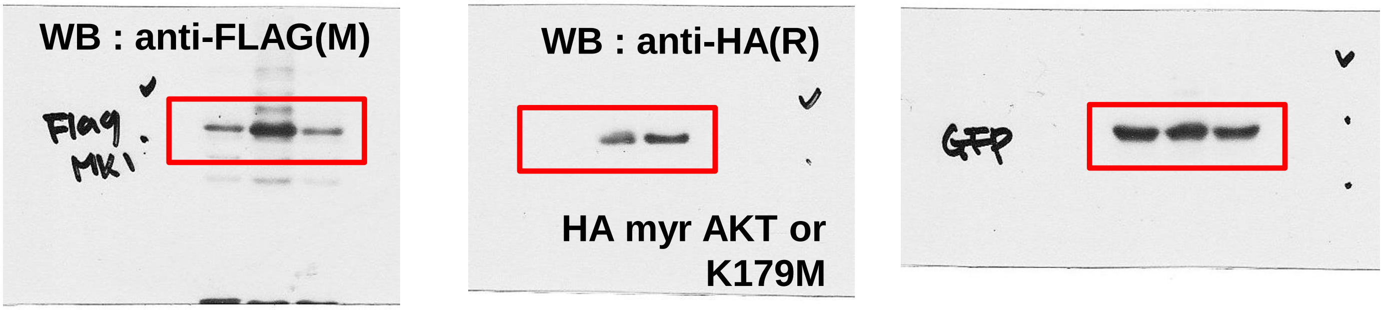

Fig. 5c

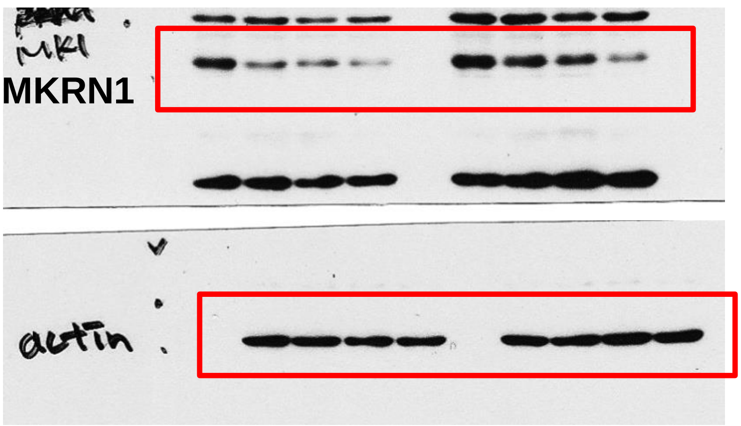

Fig. 5e

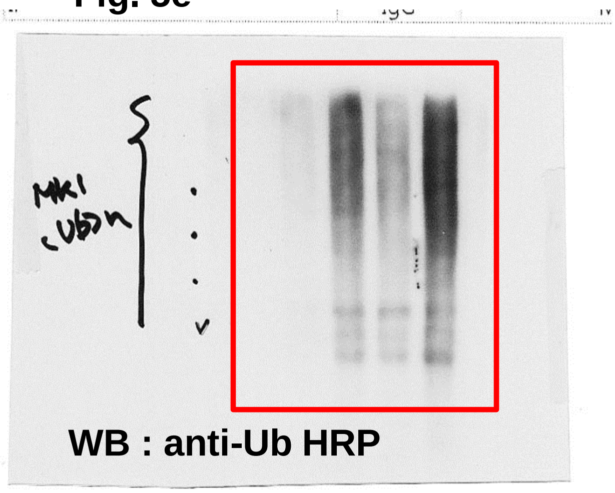

Fig. 5f

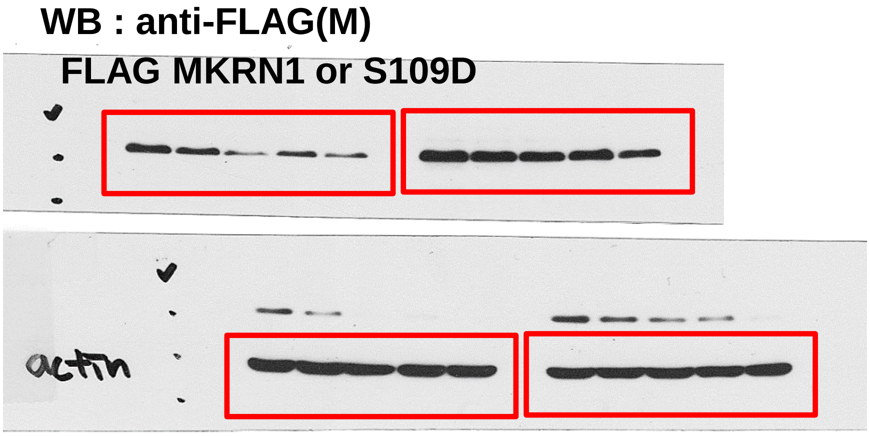

Fig. 5g

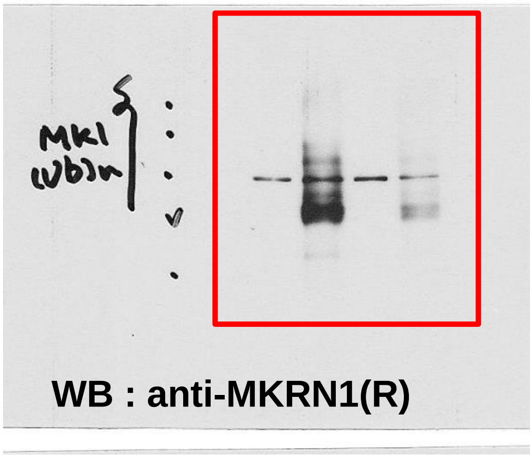

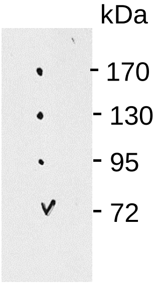

Supplementary Figure 14. Uncropped blots

(d) Blots from Figure 5.

Fig. 6a

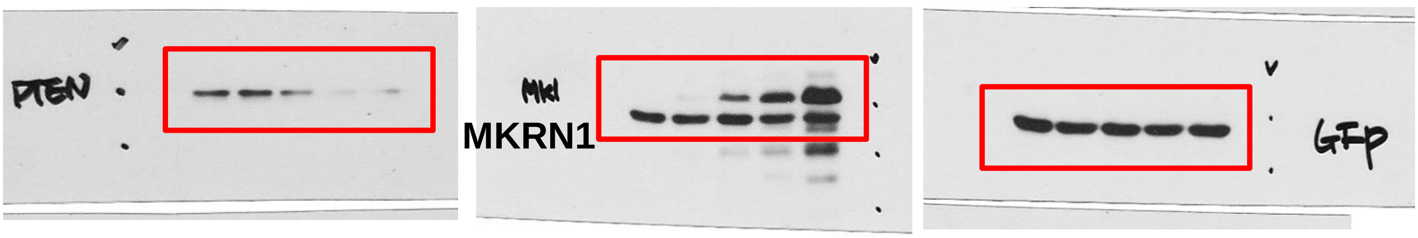

Fig. 6b

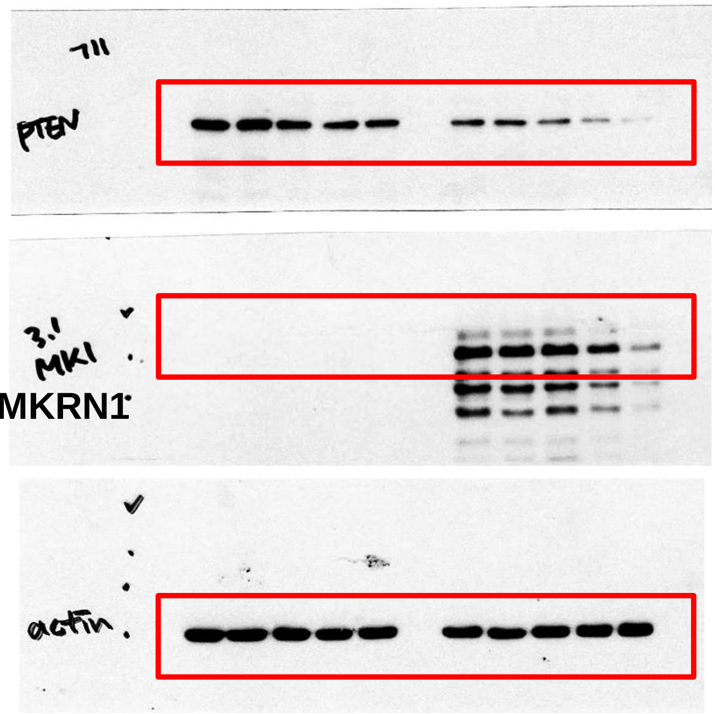

Fig. 6c

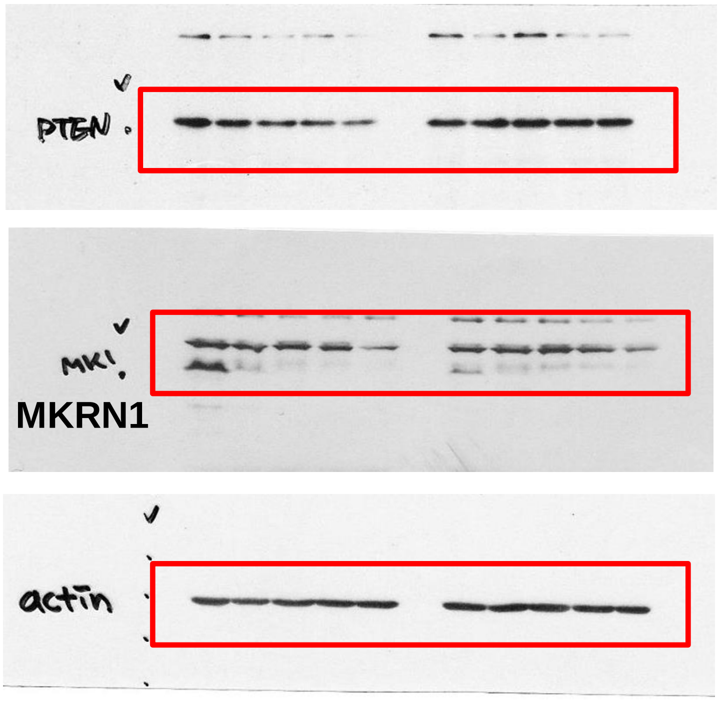

Fig. 6d

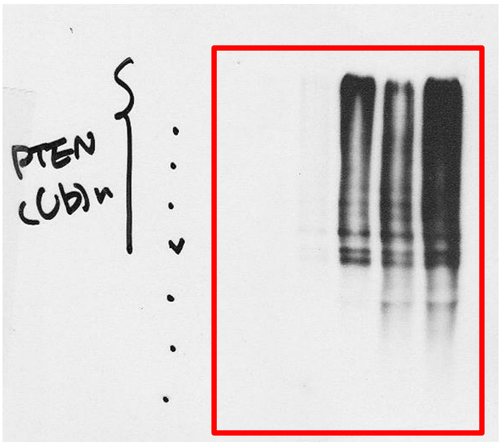

WB : anti-HA(R)

Fig. 6e

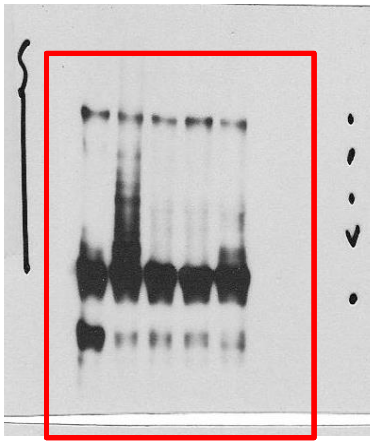

WB : anti-PTEN(M)

Fig. 6f

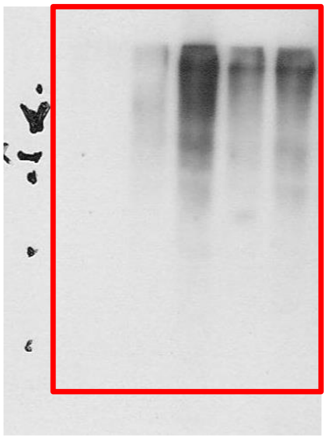

WB : anti-Ub HRP

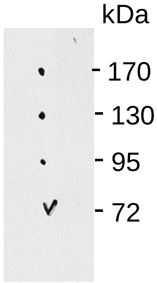

Supplementary Figure 14. Uncropped blots

(e) Blots from Figure 6.

Fig. 7a

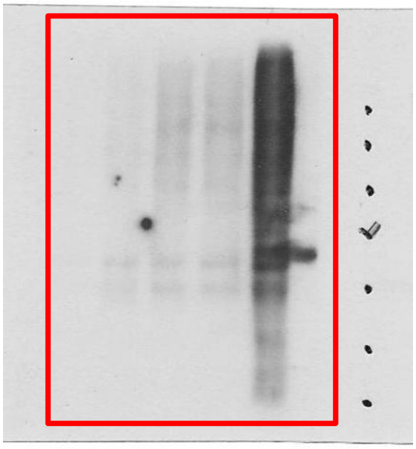

WB : anti-Ub HRP

Fig. 7b

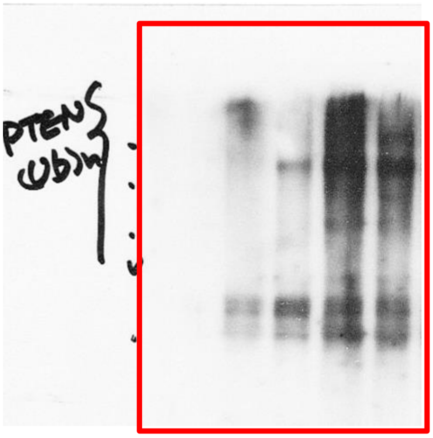

WB : anti-Ub HRP

Fig. 7c

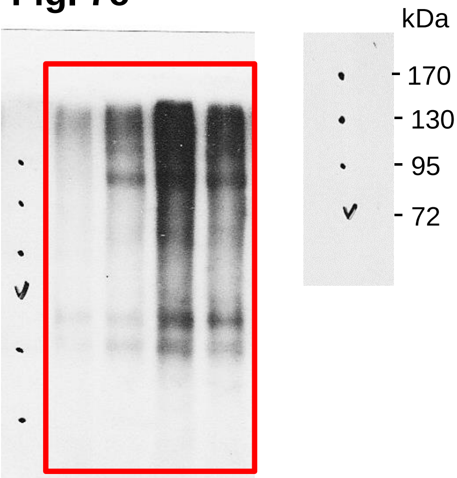

WB : anti-Ub HRP

Fig. 7e

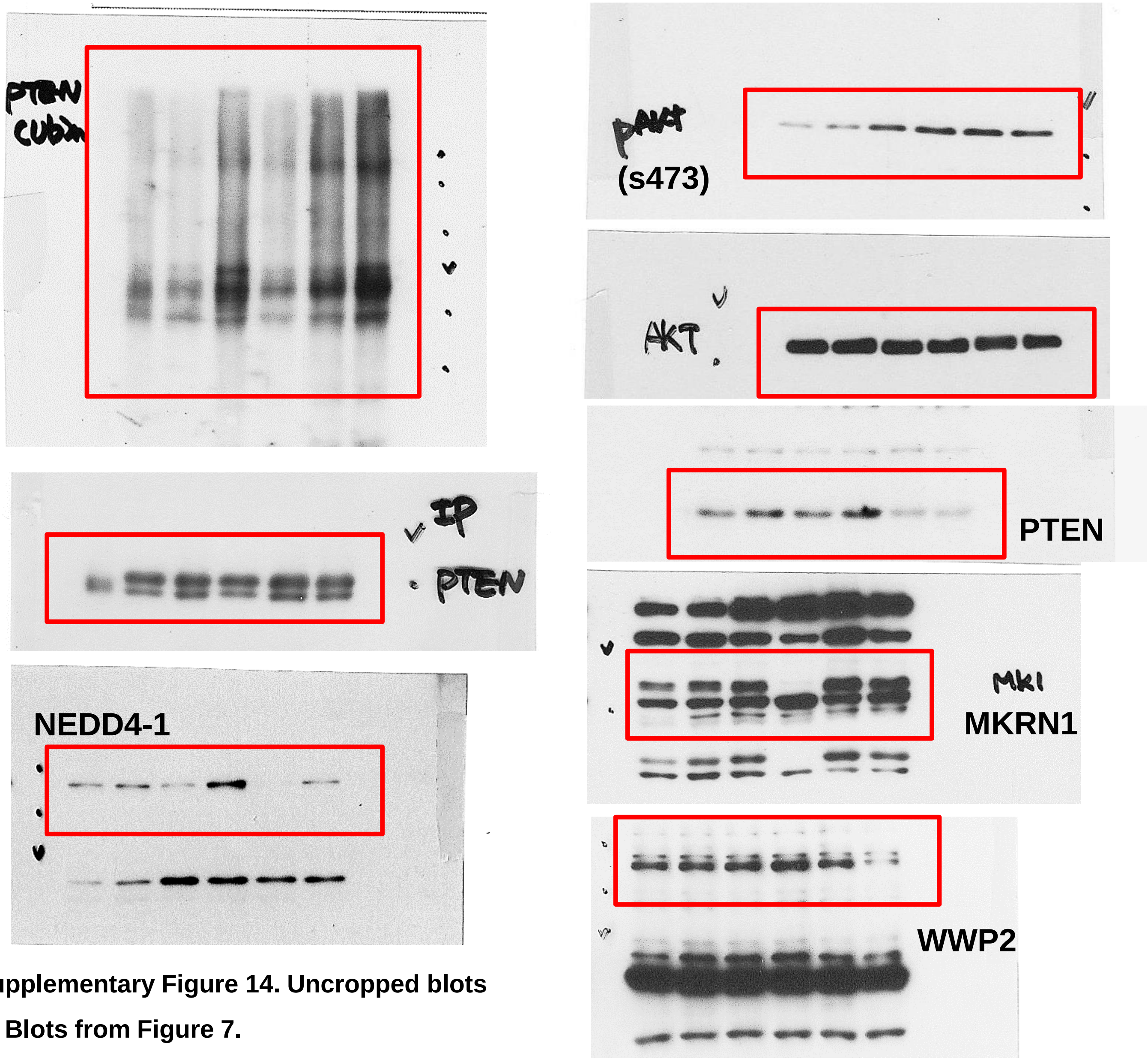

Supplementary Figure 14. Uncropped blots

(f) Blots from Figure 7.

Supplementary Tables

	MKRN1			pAKT			pmTOR			PTEN		
	N	Mean score (95% CI)	*p value*	N	Mean score (95% CI)	*p value*	N	Mean score (95% CI)	*p value*	N	Mean score (95% CI)	*p value*
**Diagnostic category**			*< 0.001*			*< 0.001*			*< 0.001*			*< 0.001*
Normal	310	2.7 (2.5-2.8)		345	2.9 (2.8-3.2)		349	1.4 (1.3-1.6)		282	3.5 (3.3-3.7)	
Low grade CIN	80	3.3 (3.1-3.6)		91	4.4 (3.9-4.9)		85	2.7 (2.3-3.1)		82	3.2 (2.9-3.6)	
High grade CIN	259	5.2 (5.0-5.5)		296	5.1 (4.8-5.3)		223	2.5 (2.3-2.7)		238	2.7 (2.5-2.9)	
Cervical cancer	169	6.4 (6.2-6.7)		181	5.5 (5.1-5.8)		165	3.6 (3.2-4.0)		181	1.2 (1.0-1.4)	
Metastasis	12	6.6 (5.7-7.5)		19	7.3 (6.0-8.7)		18	2.2 (1.8-2.7)		17	1.2 (0.6-1.9)	
**FIGO stage**			*0.018*			*0.216*			*0.613*			*0.420*
I	113	5.9 (5.6-6.3)		119	5.3 (4.9-5.7)		109	3.7 (3.3-4.2)		122	1.2 (1.0-1.4)	
II	48	6.2 (5.7-6.7)		53	6.0 (5.3-6.7)		48	3.3 (2.7-4.0)		50	1.4 (1.0-1.8)	
IV	8	7.8 (6.6-9.1)		9	5.1 (3.0-7.1)		8	3.6 (0.5-6.7)		9	0.8 (-0.3-1.9)	
**Tumor grade**			*<0.001*			*0.065*			*0.651*			*0.036*
Well+Moderate	102	5.6 (5.2-6.0)		107	5.3 (4.8-5.7)		97	3.6 (3.1-4.1)		106	1.4 (1.1-1.6)	
Poor	55	6.7 (6.3-7.2)		59	6.0 (5.3-6.7)		55	3.4 (2.7-4.2)		57	0.9 (0.6-1.3)	
**Cell Type**			*0.950*			*0.127*			*0.059*			*0.122*
SCC	140	6.1 (5.8-6.4)		148	5.3 (5.0-5.7)		136	3.8 (3.4-4.2)		149	1.3 (1.1-1.5)	
Other	29	6.1 (5.3-6.8)		33	5.3 (5.2-6.9)		29	2.8 (2.0-3.7)		32	0.9 (0.5-1.3)	
**Tumor size**			*0.625*			*0.791*			*0.745*			*0.220*
≤ 4cm	119	6.1 (5.8-6.4)		127	5.5 (5.1-5.9)		116	3.6 (3.2-4.2)		129	1.3 (1.1-1.5)	
> 4cm	50	6.0 (5.4-6.6)		54	5.4 (4.7-6.1)		49	3.5 (2.8-4.3)		52	1.0 (0.7-1.3)	
**LN metastasis**			*0.450*			*0.347*			*0.312*			*0.702*
Negative	109	6.0 (5.7-6.4)		114	5.3 (4.8-5.7)		105	3.7 (3.2-4.2)		118	1.2 (1.0-1.5)	
Positive	25	6.3 (5.4-7.3)		29	5.8 (4.8-6.7)		26	3.2 (2.0-4.3)		27	1.1 (0.6-1.7)	
**Chemoradiation**			*0.295*			*0.973*			*0.106*			*0.292*
Good response	32	5.3 (4.5-6.2)		35	5.7 (4.8-6.7)		34	3.0 (2.3-3.8)		34	1.2 (0.9-1.6)	
Bad response	8	6.3 (4.6-8.0)		11	5.8 (4.5-7.0)		7	4.6 (2.3-6.9)		9	1.6 (-0.3-1.9)	

Supplementary Table 1. Association of MKRN1, pAKT, pmTOR and PTEN IHC expression with clinocopathological characteristics in cervical cancer. Tumor sizes ranged from 0.2 to 12.0 cm (mean, 2.7 cm). The following histologic types were subjected: 156 squamous cell carcinomas (82.1%), 28 adenocarcinomas/adenosquamous carcinomas (14.7%), 5 small cell carcinomas (2.6%), and 1 clear cell carcinoma (0.6%). HC2-based HPV infection rate was 81.1% (73/90) in low grade CIN, 92.4% (220/238) in high grade CIN. The length of patient follow-up time ranged from 3 to 60 months, and median survival time at last follow-up was 38.1 months. Some data of tumor differentiation, LVSI and LN metastasis are not available on retrospective chart review. SCC, squamous cell carcinoma; LVSI, lymphovascular space invasion. Data were analyzed using a one-way ANOVA and independent t-test.

	MKRN1								
	**pAKT**			**pmTOR**			**PTEN**		
	Spearman's rho	***No. of cases***	***P value***	***Spearman's rho***	***No. of cases***	***P value***	***Spearman's rho***	***No. of cases***	***P value***
**All cancer**	0.167	169	0.030	0.065	156	0.422	-0.260	166	0.001
**Stage I**	0.207	114	0.027	0.080	103	0.424	-0.344	112	<0.001
**Stage II-IV**	0.082	55	0.551	0.052	53	0.717	-0.075	54	0.590
**SCC**	0.132	140	0.121	0.068	129	0.443	-0.234	137	0.006
**No SCC**	0.353	29	0.060	0.152	27	0.449	-0.418	29	0.024
**All CIN**	0.411	325	<0.001	0.131	279	0.029	-0.175	303	0.002
**HGCIN**	0.428	247	<0.001	0.140	200	0.048	-0.133	227	0.045
**LGCIN**	0.324	78	0.004	0.296	79	0.008	-0.020	76	0.861

Supplementary Table 2. Correlation of MKRN1, pAKT, pmTOR, or PTEN expression in cervical cancer and CIN patients. Of 190 cervical cancer specimens, 169 (88.9%) was available to confirm co-expression between MKRN1 and pAKT, 156 (82.1%) was available between MKRN1 and pmTOR, and 166 (87.3%) was available between MKRN1 and PTEN. Of 411 CIN, 325 (79.1%) was available to confirm co-expression between MKRN1 and pAKT, 279 (67.9%) was available between MKRN1 and pmTOR, and 303 (73.7%) was available between MKRN1 and PTEN. (HGCIN, high grade CIN; LGCIN, low grade CIN). Data were analyzed using a one-way ANOVA and independent t-test.
